# The Properties of Genome Conformation and Spatial Gene Interaction and Regulation Networks of Normal and Malignant Human Cell Types

**DOI:** 10.1371/journal.pone.0058793

**Published:** 2013-03-11

**Authors:** Zheng Wang, Renzhi Cao, Kristen Taylor, Aaron Briley, Charles Caldwell, Jianlin Cheng

**Affiliations:** 1 Computer Science Department, University of Missouri, Columbia, Missouri, United States of America; 2 Department of Pathology and Anatomical Sciences, School of Medicine, University of Missouri, Columbia, Missouri, United States of America; 3 Informatics Institute, University of Missouri, Columbia, Missouri, United States of America; 4 Christopher S. Bond Life Science Center, University of Missouri, Columbia, Missouri, United States of America; University of Geneva, Switzerland

## Abstract

The spatial conformation of a genome plays an important role in the long-range regulation of genome-wide gene expression and methylation, but has not been extensively studied due to lack of genome conformation data. The recently developed chromosome conformation capturing techniques such as the Hi-C method empowered by next generation sequencing can generate unbiased, large-scale, high-resolution chromosomal interaction (contact) data, providing an unprecedented opportunity to investigate the spatial structure of a genome and its applications in gene regulation, genomics, epigenetics, and cell biology. In this work, we conducted a comprehensive, large-scale computational analysis of this new stream of genome conformation data generated for three different human leukemia cells or cell lines by the Hi-C technique. We developed and applied a set of bioinformatics methods to reliably generate spatial chromosomal contacts from high-throughput sequencing data and to effectively use them to study the properties of the genome structures in one-dimension (1D) and two-dimension (2D). Our analysis demonstrates that Hi-C data can be effectively applied to study tissue-specific genome conformation, chromosome-chromosome interaction, chromosomal translocations, and spatial gene-gene interaction and regulation in a three-dimensional genome of primary tumor cells. Particularly, for the first time, we constructed genome-scale spatial gene-gene interaction network, transcription factor binding site (TFBS) – TFBS interaction network, and TFBS-gene interaction network from chromosomal contact information. Remarkably, all these networks possess the properties of scale-free modular networks.

## Introduction

A genome of a cell is a complete collection of double-stranded linear DNA sequences of a species. It contains protein coding regions (i.e., gene), gene regulatory elements (e.g., promoter and enhancer), and non-coding functional or nonfunctional regions (e.g., microRNA and intron). The genome encodes all the genetic information necessary for a cell to function throughout its life cycle. The cell of a eukaryotic species forms a multi-granularity genome structure (e.g., nucleosome, chromatin fiber, chromatin cluster, chromosome, and genome) in order to compactly store a very long genomic DNA sequence in its small nucleus. A nucleosome is a basic unit consisting of ∼145–147 base pairs of DNA wrapped around a protein complex (histone octamer). Tens of nucleosomes are further collapsed into a larger dense structural unit – chromatin fiber – of several kilobase (Kb) pairs [1], [2]. Multiple chromatin fibers form a large module of megabase pairs (Mb) DNA, which may be referred to as domains, globules, gene loci, or chromatin clusters in different contexts. A number of chromatin clusters then fold into a large independent physical structure – chromosome [3], [4], which occupies a physical space in nucleus often being referred to as chromosome territory [5], [6]. One or more chromosomes interact to constitute the dynamic three-dimensional (3D) conformation of the entire genome of a cell.

Examination of the spatial conformation of a genome is essential for understanding long-range gene-gene interaction, spatial gene regulation, DNA methylation, and chromatin remodeling that involve linearly distant genes and functional elements of several kilobase or even megabase nucleotides away on a linear genome [5]–[10]. In contrast to the extensive research on genome-wide gene expression and DNA methylation in a linear genome facilitated by whole genome sequencing, the detailed investigation of the spatial conformation of a genome has just been enabled by several recently invented chromosome conformation capturing methods (e.g., 3C, 4C, and 5C) that can interrogate genome structure at a large scale [7], [8]. Different from an early, but still widely used method, fluorescence in-situ hybridization (FISH) [9] that can selectively measure the physical distances between a number of genetic markers (e.g., a marked position on a chromosome), 3C [10], 4C [11], and 5C [12] methods empowered by DNA sequencing techniques, have determined chromosomal regions in spatial proximity (or contact) within a pre-marked genomic region of up to a few Mb. More recently, the Hi-C technique [13] empowered by next generation sequencing was designed to determine both intra- and inter- chromosomal contacts in an unbiased manner at the whole genome scale. The Hi-C technique joins together the spatially close, but linearly separated genome fragments by ligation, and then excises the combined fragments off for DNA sequencing. The two parts of the combined sequences are then mapped to a reference genome (e.g., human genome in this work) in order to identify the genomic regions or locations that are in spatial proximity – contact. The Hi-C technique can determine chromosomal contacts with higher resolution by increasing the depth and coverage of sequencing. Thanks to the wide availability of next generation sequencing facilities and the standard protocol of preparing Hi-C libraries, the Hi-C technique is poised to be widely used to generate chromosomal contact data for studying spatial genome conformation at either 2D or 3D levels in order to elucidate its role in gene interaction, gene regulation, and DNA methylation [14]–[16]. Accordingly, computational methods need to be developed to generate, analyze, and model these new sources of data in a large-scale manner in order to study the structural and functional properties of a genome in the spatial context.

In this work, we generated hundreds of millions of Hi-C paired-end sequence reads for three different human cells (RL follicular lymphoma cell line, primary tumor B-cells from an acute lymphoblastic leukemia patient, and MHH-CALL-4 B-cell acute lymphoblastic leukemia cell line) using the Hi-C technique. An in-house bioinformatics software pipeline was developed and applied to map sequence reads to the human reference genome, producing a large data set of high-quality and high-resolution chromosome contacts. Our computational analysis on these data reveal some interesting properties of human genome conformation, including conformational conservation and variation of genomes of different cells, intra- and inter-chromosomal interactions, aberrant chromosomal translocation, spatial gene clusters, spatial gene-gene interactions, and spatial gene-regulatory-element interaction. Furthermore, we derived spatial interactions between functional elements (genes, transcription factor binding sites) from the chromosomal interaction data. The data were then used to generate chromosome-/genome-wide gene-gene interaction networks, transcription factor binding site (TFBS) – TFBS networks, and gene-TFBS networks. Remarkably, the connectivity in both networks shows the hallmark features of scale-free networks, suggesting that spatial interactions of gene-gene, gene-TFBS, and TFBS-TFBS in a genome are far from random. These findings may lead to new biological insights into spatial gene-gene interaction and regulation. And in contrast to previous studies investigating the organizer role of human CCCTC-binding factor [17] and the binding patterns of 45 human transcription factors [18], our networks provide a large-scale study of gene and TFBS interactions at the chromosome/genome level and reveal their scale-free interaction patterns. Our gene-gene, gene-TFBS, and TFBS-TFBS networks may also provide a graphic way of studying the functional relationship between interacted genes and TFBS [19].

## Results

### Hi-C read mapping

We created Hi-C libraries for a case of primary human B-acute lymphoblastic leukemia (B-ALL), the MHH-CALL-4 B-ALL cell line (CALL4), and the follicular lymphoma cell-line (RL). These libraries were sequenced using an Illumina HiSeq 2000. High-quality paired-end reads of 39M, 79M, and 33M were obtained for these cells, respectively. The quality distributions of 100–120 bp reads are shown in **Figures S1** **, S2, and S3**. The read number distributions and gene number distributions along selected chromosomes are reported in **Figure S4**. The paired-end DNA-reads of the normal human B-cell line (GM06990) (7M pair of reads) were downloaded from Lieberman-Aiden etc [13] as a reference benchmark to test our in-house read mapping method. Paired-end reads for both the reference data and our own Hi-C data were mapped to the human genome and the chromosomal contact information was generated (see details in “Methods” section). On the reference data, 98.3% of the contacts generated by our method were identical with the contacts produced in [13]; and 83.2% of contacts in [13] were also reproduced by our method. This high consistency supported the validity of our system. The reads that were mapped to multiple locations in the genome were discarded. In terms of sequencing depth, on average, each gene region in our Primary ALL, MHH-CALL-4, RL cell lines has about 1.8, 2.8, and 1.5 mapped reads (**Table S1**), which is much higher than 0.17 in [13] likely because of our higher level of sequencing reads.

### Intra-chromosomal contacts of different cells/cell lines

**Figure 1:**
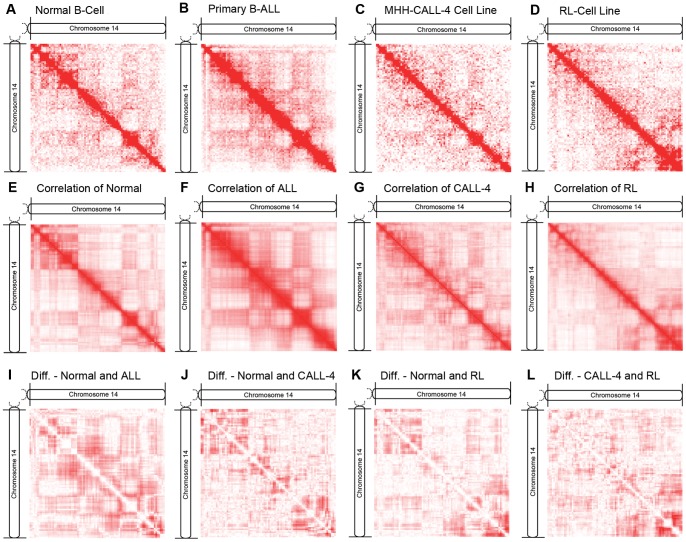
The contact matrices, correlation matrices, and difference matrices of chromosome 14. This figure illustrates the original contact matrices, correlation matrices, and difference matrices of chromosome 14 for the normal human B-cell, human acute lymphoblastic leukemia B-cell, human MHH-CALL-4 B-ALL cell line, and human lymphoma RL cell-line. Heat maps (A-D) visualize the original number of contacts within the chromosome, (**E**-**H**) the Pearson’s correlation matrices generated from the original contact matrices, and (**I**-**L**) the absolute difference matrices generated from the correlation matrices.

We constructed the intra-chromosomal contact matrices with and without normalization for the normal B-cell, primary B-ALL cells, MHH-CALL-4 cell line, and lymphoma RL cell line. The contact matrices without normalization were visualized as heat maps (**Figure** **1A–D** for chromosome 14, **Fig S5, S6, S7, S8, S9, S10, S11, and S12** for all chromosomes with and without normalization). In a heat map matrix (*M*), a chromosome is divided into a number of 1 Mb regions, where the value of a cell *M_i,j_* is the number of contacts between regions *i*, and *j*. Although 1Mb resolution contact matrices were generated here for comparison with the reference data [13], higher resolution matrices/maps (e.g. 10 Kb resolution) can also be generated for our cell samples or at least some chromosomal regions since many more reads were collected. In the heat maps of visualizing contact matrices, the intensity of color at position *i*, *j* is set proportional to the contact frequency between two regions *i*, *j*. The heat maps we constructed for the reference normal B-cell (**Figure** **1A**) are almost identical to those in [13], supporting the validity of our method. As in [13], in order to sharpen contact patterns, we generated Pearson’s correlation maps (*C*) from initial contact maps (**Figure** **1E–H**), in which the value of a cell *C_i, j_* was equal to the Pearson’s correlation between the *i*th and *j*th rows in the original contact matrix (*M*). The assumption is that if two regions are spatially close, they should share similar contact neighbors, thus have a higher correlation between their contact profiles. Indeed, the correlation maps clearly reveal the plaid contact patterns likely corresponding to open/closed euchromatin and heterochromatin compartments than in the initial contact matrices by reducing noise in the data. The results suggest that the correlation maps of the normal B-cell, primary B-ALL, and CALL4 (B-ALL cell line) (**Figure** **1E–G**) are more similar to each other than to the RL cell line (follicular lymphoma cell line) (**Figure** **1H**), even though there are also some differences in the maps of normal and malignant B-cells (**Figure** **1E–F**). To reveal the differences in contact profiles between healthy and malignant B-cells and between different cell types, we constructed difference matrices showing the absolute difference between their correlation maps. **Figure** **1I–K** illustrates the differences in the correlation maps of chromosome 14 between normal B-cells and the primary B-ALL cells, MHH-CALL-4 cell line, and lymphoma RL cell line, respectively. **Figure** **1L** shows the difference between two malignant cell lines MHH-CALL-4 and follicular lymphoma RL. Higher color intensity indicates larger absolute difference and white indicates the same. It seems that the difference between the normal and malignant B-cells is more obvious than that between the other pairs. The Pearson’s correlations between the vectors of intra-chromosomal contact numbers of 23 pairs of chromosomes of these four samples (**Fig. S13**) also show similar relationships. **Fig. S14** shows the significance analysis of intra-chromosomal contact matrices.

**Figure 2:**
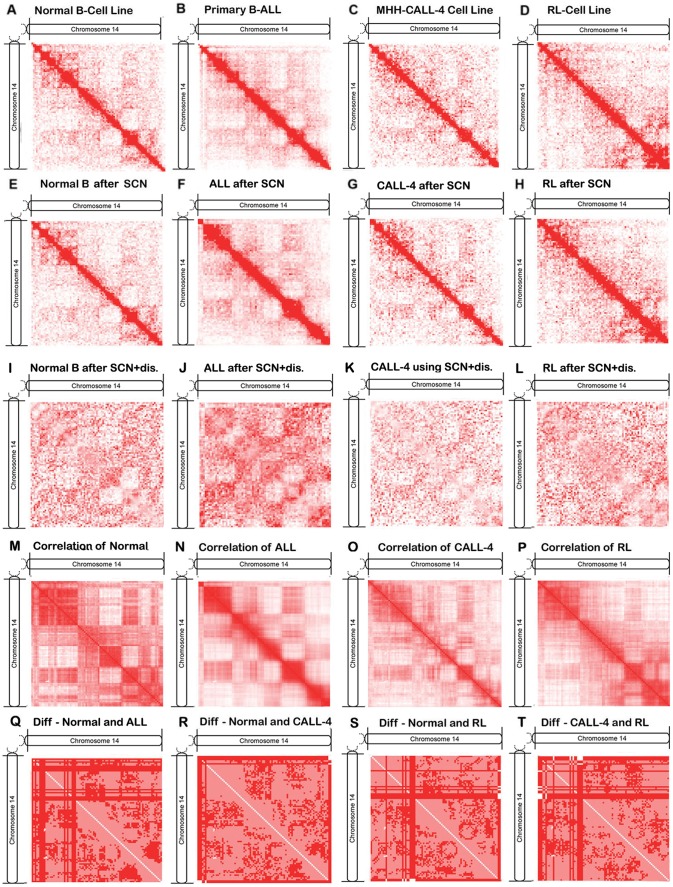
The contact matrices, correlation matrices, and difference matrices of chromosome 14 after applying normalization. (**A–D**) denote the contact matrices after removing reads mapped to multiple locations on the genome, (**E-H**) the contact matrices after applying SCN normalization method to the maps in (**A–D**), (**I–L**) the contact matrices after applying both genomic sequential distance based method and SCN to maps in (**A–D**), (**M–P**) the correlation matrices based on maps in E-H, and (**Q–T**) the difference matrices of between correlation matrices in M-P of normal and malignant samples.

In contrast, we generated another set of contact matrices (**Figure** **2**) with normalization. **Figures** **2A–D** denote the raw contact matrices derived from the reads mapped to the unique locations in the genome. **Figures** **2E–H** visualize the contact matrices generated by further normalizing the raw contact matrices using Sequential Component Normalization method (SCN) [19]. Furthermore, in order to emphasize long-range interactions as in [20], we first use a genomic sequential-distance-based normalization method [19] to normalize the raw contact maps and then apply the SCN method to them to generated the contact maps visualized in **Figure** **2I–L**, which deemphasizes the large number local contacts in the diagonal regions in the contact maps. **Figure 2M–P** show the correlation maps based on the normalized contact maps in **Figure 2E–H**. Compared with the correlation maps generated based on raw contact numbers in **Figure** **1A–D**, the contact normalization indeed makes some parts of the patterns clearer while keeping the overall contact profile similar. **Figures** **2Q–T** show the differences between healthy and malignant correlation maps that were generated from the normalized contact maps in **Figure** **2A–D**. The dark red indicates the sign of correlations was changed (i.e., from positive correlation value to negative or vice versa) in two cell types. Light red denotes a change in correlation value without sign change. White indicates correlation is not changed. It is shown regions with sign changes from one cell type to another tend to be adjacent or form some rectangular patterns. Further studies are needed to explain their functional and structural implication.

### Inter-chromosomal contacts and chromosomal translocations

Inter-chromosomal contacts exist between two spatially close regions from two different chromosomes. We generated normalized inter-chromosomal contact matrices for all pairs of chromosomes for all cellular samples (see **Fig. S15** for an example between chromosome 11 and 14 for Normal B-Cell Line). In comparison with plaid patterns in intra-chromosomal contact maps, inter-chromosomal contacts are much less frequent and more uniformly distributed in all but one case. The unusually dense inter-chromosomal contacts were found between chromosomes 11 and 14 for the primary B-ALL cells (data not shown) suggesting the telomeric end of chromosome 11 is very spatially close to chromosome 14 and a part of chromosome 14 is unusually close to chromosome 11. The most plausible explanation for this is that a reciprocal translocation of chromosome 11 and chromosome 14 was present because the unusually dense “inter-”chromosomal contacts actually were “intra-”chromosomal contacts caused by translocation. By zooming in on the inter-chromosomal contact map from 1Mb resolution to 10Kb, the boundaries of the translocation between chromosomes 11 and 14 were identified computationally by detecting the locations with a sudden and drastic increase of inter-chromosomal contacts (**Fig. S16**). The translocation was later confirmed by an independent oligonucleotide tiling array experiment specialized at detecting chromosome rearrangements and was shown to be a cancer causing factor (manuscript in preparation). Based on the translocation, our method was able to reconstruct the translocated chromosomes 11 and 14 (t;11:14), and to estimate their inter-chromosomal contacts (**Fig. S17)**.

**Figure 3:**
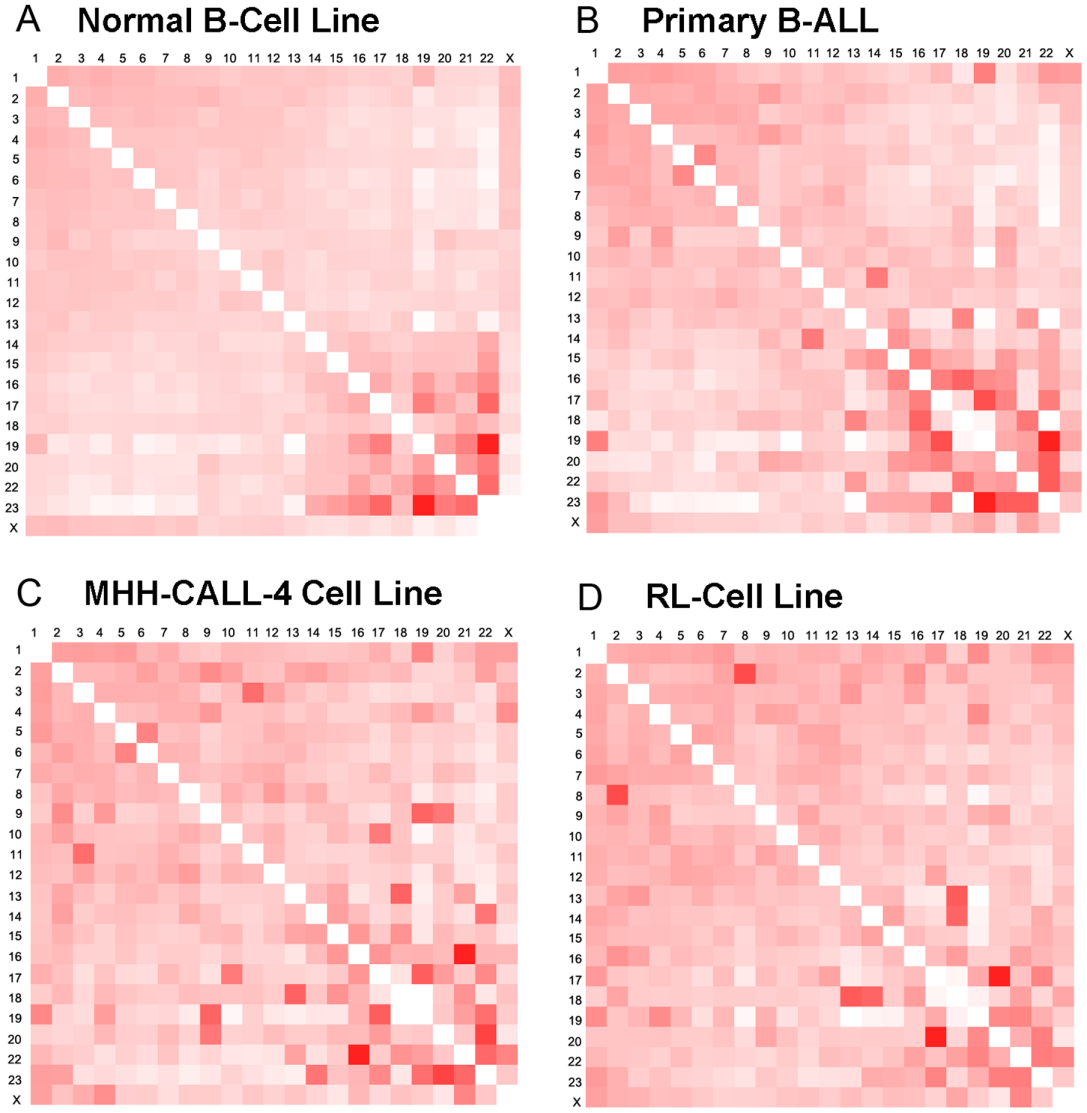
The characteristics of inter-chromosomal contacts. This figure shows the observed numbers of inter-chromosomal contacts divided by the expected numbers of inter-chromosomal contacts between all pairs of human chromosomes.

In order to study the genome-wide inter-chromosomal contact profiles between healthy and malignant cells and between different cell types, we calculated the ratio between the observed and the expected number of contacts for all pairs of human chromosomes for the normal B-cell, primary B-ALL cells, the MHH-CALL-4 cell line, and the follicular lymphoma RL cell-line. The higher ratios indicate more enrichment of inter-chromosomal contacts between two chromosomes. Our genome-wide inter-chromosomal contact profile generated on normal B-cell is highly similar to the one in [13], in which small gene-rich chromosomes have more interactions than large gene-sparse chromosomes. While this is still largely true for primary malignant B-ALL cells and the MHH-CALL-4 cell line (data not shown), unusual dense inter-chromosomal contacts were also found occurring in large or small chromosomes in these cells/cell lines. For example, in comparison with the normal B-cells, more contacts were found between chromosome 1 and chromosome 19, chromosome 11 and 14 in primary B-ALL tumor cells (**Figure** **3B**); chromosome 5 and chromosome 6, chromosome 3 and chromosome 11, chromosome 10 and 17, chromosome 9 and chromosome 19, chromosome 16 and chromosome 21 in the MHH-CALL-4 cell line (**Figure** **3C**). Particularly, there are unexpectedly more contacts between chromosome 2 and chromosome 8, chromosome 17 and chromosome 20, chromosome 13 and chromosome 18 in the RL-cell line (**Figure** **3D**) compared with normal B-cells. Moreover, in comparison with the normal B-cells, the small and gene-rich chromosomes appear to have fewer contacts in the lymphoma sample (**Figure** **3D**). The Pearson’s correlations between total numbers of inter-chromosomal contacts of four cells/cell lines are reported (**Fig. S18**). The difference in inter-chromosomal interaction patterns may shed light on the biology of different cell types and potential causes of diseases.

In addition to the conservation and variation in inter-chromosomal interactions described above, one remarkable conserved interaction among all four cells/cell lines is that one region [18Mb, 19Mb] in chromosome 14 always has the maximum number of inter-chromosomal contacts with other chromosomes. The most enriched gene function of the genes in this region is *ubiquitin-protein ligase activity* (Gene Ontology term, GO:0004842), but the importance of this function awaits further investigation.

Furthermore, in order to illustrate the importance of removing the noise in the data, we calculated the proportion of inter- and intra-chromosome interactions before and after using the SCN normalization procedure [21] (see **Fig. S19**). The number of inter- and intra-chromosome interactions before using the SCN normalization procedure is calculated by counting the total number of them from contact file directly. The number of inter- and intra-chromosome interactions after using the SCN normalization procedure is calculated from the normalized inter- and intra- contact matrices. It is shown that the proportion of inter-chromosome interactions is reduced after using the SCN normalization procedure.

### Chromosome-/genome-wide spatial gene-gene interaction networks

**Figure 4:**
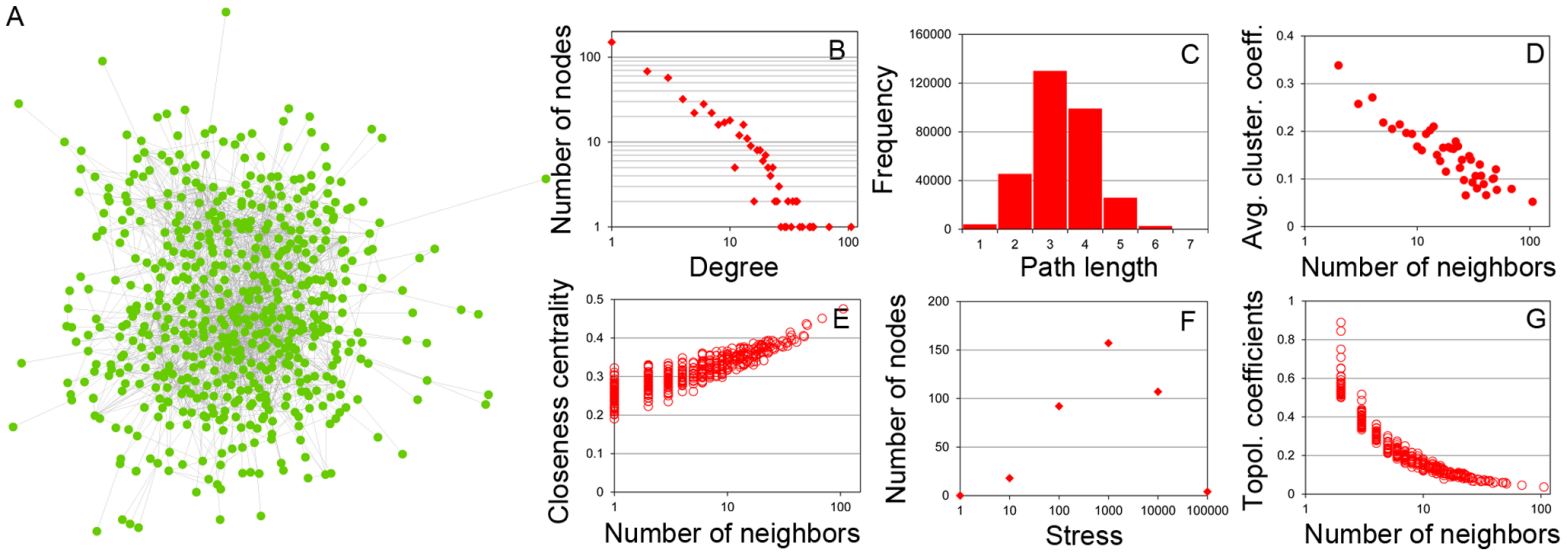
The spatial gene-gene interaction networks and the analysis of its properties. The results were based on the contact data with reads that were mapped to multiple locations on the genome removed. (**A**) The gene-gene interaction network of genes residing in chromosome 14 for the CALL-4 cell line. (**B**) The distribution of node degrees. (**C**) The histogram of shortest path lengths. (**D**) The plot of average clustering coefficient against of the degree (the number of neighbors) of a node. (**E**) The plot of closeness centralities against the degree of a node. (**F**) The stress distribution. (**G**) The plot of topological coefficients against the degree of a node. The network and its properties were visualized and analyzed by Cytoscape [32].

We chose the *HoxA* gene cluster [14] in chromosome 7 (location 27,095,000 – 27,215,000) to compare interaction patterns of different cell lines in greater detail (**Fig. S20**). **Fig. S20** shows the non-normalized absolute number of contacts for *HoxA* genes, which may contain noise partially due to the potential low reads coverage in a small genome region. **Fig. S21** shows the reads mapped to this cluster visualized by the UCSC Genome Browser [22]. The large amount of Hi-C chromosomal interaction data provided an unprecedented opportunity to study spatial gene-gene interactions. To the best of our knowledge, for the first time, we constructed chromosome-/genome-wide spatial gene-gene interaction networks for the human genome. In these networks, each node represents a gene; and an edge is used to connect two genes if there is at least one Hi-C read showing they are in spatial contact. The weight of the edge is the number of observed contacts between two genes. **Figure** **4A** illustrates the intra-chromosomal gene-gene interaction network of the genes on chromosome 14 for the MHH-CALL-4 cell line. The isolated genes without any spatial contact with other genes and the genes with types of “PSEUDO”, “RNA”, “CDS”, and “UTR” were not included. We analyzed a number of properties of the network, including node degree distribution (**Figure** **4B**), shortest path length distribution (**Figure** **4C**), average clustering coefficient distribution (**Figure** **4D**), closeness centrality (**Figure** **4E**), stress distribution (**Figure** **4F**), and topological coefficients (**Figure** **4G**). The linear relationship in the log-log plot of node-degree distribution (**Figure** **4B**) shows that the gene-gene interaction network is very likely a scale-free network, like human social networks, world wide web, protein-protein interaction networks [23]–[27], and protein domain co-occurrence networks [28], where most nodes have few connections and some hub nodes have many connections. For example, a hub gene (GeneID:9369) has interactions with 111 other genes in chromosome 14. The gene-gene interaction networks of several chromosomes of other cells/cell lines that we investigated also possess the same scale-free property (data not shown). **Figure** **4C** further depicts the small-world phenomenon [23] associated with a scale-free network, i.e. most of genes are three or four steps away from each other through the shortest path between them. **Figure** **4D** shows that the hub nodes with many interactions tend to have small clustering coefficients, whereas nodes with fewer connections tend to cluster with others to form densely connected sub-graphs. These clusters are connected through hub nodes. We also found that the nodes with smaller degree have the less average closeness centrality that measures how fast information can be spread to other reachable nodes, whereas hub nodes usually have a higher closeness centrality, which might suggest their importance in maintaining the connectivity of the networks (**Figure** **4E**).

The stress value of a node is the number of shortest paths passing through it. A higher stress value may imply a more important role of the node in the network. A small portion of nodes have a very high or very low stress value and most nodes have a middle stress value (**Figure** **4F**), indicating that a small number of nodes may be extremely important to the network. The topological coefficient of a node measures the extent to which it shares neighbors with others. **Figure** **4G** shows that hub nodes usually tend not to share neighbors. Instead, they serve as a center of a group of nodes (i.e. a module or clique), and the modules are connected through their hub nodes indirectly.

The inter chromosome gene-gene networks are generated for comparison, and we also find out the scale free network properties for them (**Fig. S22, S23, S24, and S25** shows an example of inter chromosome gene-gene networks between chromosome 11 and 14 for Primary B-ALL).

### Interaction networks of transcription factor binding sites (TFBS)

In order to study interactions between TFBS that play an important role in spatial gene regulation, we constructed interaction networks of transcription factor binding sites (TFBS) from the Hi-C data. The whole-genome TFBS-TFBS interaction network for the MHH-CALL-4 cell line is shown in **Fig. S26**. Like spatial gene-gene interaction networks, the TFBS-TFBS interaction networks also have the hallmark features of scale-free networks (**Fig. S27, S28, S29, and S30**). This *de novo* network may help study how spatially distal genes may be brought together to share the same transcription machinery. For comparison, we also generate one normalized TFBS-TFBS interaction networks between chromosomal 4 and chromosomal 4 of the MHH-CALL-4 cell line (**Fig. S31, S32, and S33**).

### Interaction networks of transcription factor binding sites and genes

To investigate interactions among TFBS and genes, we constructed networks showing TFBS-TFBS-gene interaction relationship for chromosome 14 of the MHH-CALL-4 cell line (**Fig. S34**). The statistical properties of TFBS-TFBS-gene interaction network suggest it is a scale-free network (**Fig. S35, S36, S37, and S38**) that is very different from a random network. One TFBS on chromosome 14 that has a lot of contacts with a gene (GeneID: 145508) along its location was visualized by the UCSC Genome Browser (**Fig. S39)**.

## Discussion

We developed a bioinformatics pipeline to study the properties of human genome conformation and spatial gene interaction and regulation networks by analyzing Hi-C data. Our computational method can reliably generate intra- and inter-chromosomal contact matrices according to a standard Hi-C benchmark [13]. The chromosomal contact matrices built on the Hi-C data of three malignant cells/cell lines (B-ALL CALL-4, and RL) and a normal B-cell line demonstrates both the conservation in the genome conformation across different cells and the cell-type-specific or cell-state-(i.e. disease versus normal)-specific variation. For instance, smaller chromosomes had more contacts than expected compared with large chromosomes in the normal B-cell, but the pattern became less obvious in the malignant B-cells/cell lines, especially in the follicular lymphoma RL cell line. The conformational difference between different cell lines may be used to help understand 3D nuclear conformation and disease associations in different malignancies.

Furthermore, our computational analysis on high-resolution (e.g. 10Kb) inter-chromosomal contact matrix built on a case of primary B-ALL successfully identified a cancer-related chromosomal translocation between chromosomes 11 and 14 based on abnormal intensive interactions between the two ends of the two chromosomes. The boundaries of the translocation were accurately detected and then used to construct the *in silico* translocated chromosomes. This is probably one of the first few examples that the Hi-C method can be used to accurately pinpoint and re-construct clinically important chromosomal translocations.

In addition to studying the properties of the genome and chromosome conformation as a whole, we also developed methods to use Hi-C data to investigate both the gene-gene interactions in the *HoxA* gene cluster on chromosome 11 and the chromosome-wide spatial gene-gene interactions. To the best of our knowledge, this is the first demonstration of chromosome-/genome-wide spatial interaction networks between genes and transcription-factor-binding-sites (TFBS). Our experiments show that these gene interaction and regulation networks have the properties of modular scale-free networks similar to other biological networks. These discoveries shed new light on the study of 3D nuclear gene interactions and regulation.

## Methods

### Hi-C library preparation and sequencing of the primary human acute lymphoblastic leukemia B-cell (B-ALL), MHH-CALL-4 B-ALL cell line (CALL4), and lymphoma RL cell-line (RL)

The primary ALL patient sample was obtained from the Ellis Fischel Cancer Center (Columbia, MO) following diagnostic evaluation and in compliance with the local Institutional Review Board. Human cell lines RL and MHH-CALL4 were maintained at 37°C with 5% CO_2_. MHH-CALL4 is a human B cell precursor leukemia cell line established from the peripheral blood of a 10-year-old Caucasian boy with acute lymphoblastic leukemia at diagnosis in 1993 [29].

Library preparation was adapted from [13]. Briefly, cells from a patient with acute lymphoblastic leukemia, a lymphoma cell line (RL), and an acute lymphoblastic leukemia cell line (MHH-CALL4) were cross linked by adding 1.25mL of 37% formaldehyde to a final concentration of 1%. After a 10 minute incubation, 2.5 mL of 2.5M glycine was added to stop the reaction. Once cross-linked, the cells were lysed and the chromatin was digested by adding 400 units of HindIII and incubating overnight at 37°C with rotation. The ends of the fragmented DNA were repaired by adding 1.5 µL 10 mM dATP, 1.5 µL 10 mM dGTP, 1.5 µL 10 mM dTTP, 37.5 µL 0.4 mM biotin-14-dCTP, and 10 µL 5 units/µL Klenow and incubating at 37^o^C for 45 minutes. For dilute blunt-end ligation 7.61 mL of ligation mix (745 µL of 10% Triton X-100, 745 µL 10x ligation buffer (500 mM Tris-HCl pH7 5, 100 mM MgCl2, 100 mM DTT), 80 µL 10 mg/mL BSA, 80 µL 100 mM ATP and 5.96 of water) was added and incubated for 6 hours in a circulating water bath at 16°C.This process marked the DNA with biotin and an NheI recognition sequence was formed at the ligation junction. The DNA was then purified by degrading the remaining proteins with proteinase K and performing phenol-chloroform extractions. The detailed description of the entire Hi-C procedure used in our experiment is described in the supplemental document **Text S1**.

To verify ligation efficiency, PCR was performed using quality control primers (Lieberman et al.). The PCR products were then digested with HindIII or NheI. As in [13], we detected an approximate 70% ligation efficiency in our Hi-C libraries. **Fig. S40 and S41** show the ligation efficiency and PCR digest control of a few cell samples.

After the ligation efficiency was validated, biotin was removed from unligated DNA fragments using T4 DNA polymerase. The DNA was then sheared to a size of 300–500 base pairs and streptavidin beads were used to collect the remaining biotin labeled DNA fragments. The three Hi-C libraries were then subjected to paired-end high-throughput sequencing on the Illumina HiSeq 2000 by core facilities at the University of Missouri-Columbia.

### Mapping Hi-C sequence reads to the reference genome

The sequencing generated millions of pair-end reads for each sample above. Each read end has a length of 100 or 120 nucleotides (**Table S1 and S2**). The Hi-C sequence reads of the normal B-cell line were downloaded from [13] to test and ensure the correctness of our methods. The sequence reads of the four cell lines were mapped to the human genome according to the protocol illustrated in **Fig. S42**. Briefly speaking, software Maq was used to map each read-pair to the reference human genomes (NCBI build 36.3), where parameter “sum of mismatching base qualities (-e)” controlling the tolerance of mismatches was set to 150 in most experiments. Maq outputs the base pair positions in the reference genomes where each DNA read is mapped to. The mapped positions were analyzed by our method to generate chromosomal contacts. Although one read may be mapped to multiple locations due to inexact match of Maq, only the reads mapped to a unique location were kept for generating the contacts. This strict strategy of handling multiple mapping locations reduced noise in the data and ensured the high quality of contacts. Moreover, we only kept the reads-pairs whose two ends are either mapped to two different chromosomes or > = 2K bp away on the same chromosome (**Fig. S42**). As an example, the reads mapped to the HoxA gene cluster region in chromosome 7 were visualized in **Fig. S21**.

### Normalization for Hi-C contact maps and gene and transcription factor binding sites networks

There are various bias and background noise in Hi-C raw data, such as the bias of fragments sizes, GC content and circularization steps. So it is important to normalize the Hi-C data before analyzing them [15], [20], [30]. In this paper, the Sequential Component Normalization (SCN) [20] normalization procedure was used to normalize both intra and inter chromosome contact maps as follows. For a contact matrix, each column is considered as a vector and each element in the vector is divided by its Euclidean Norm. And then, each row of the contact matrix is normalized in the same way. The two steps of the procedure above are repeated until the contact matrix becomes symmetric. Since the SCN normalization tends to give an equal weight to each region in the contact map, generally regions with very low number of reads are removed before the SCN normalization is applied. Figures S43 demonstrates the distribution of Euclidean Norms of each region of both inter and intra chromosome contact maps for MHH-CALL-4. For intra chromosome contact maps, another step is added to take into account the effect of the genomic distance, that is the contact number of two regions in the intra contact matrix is divided by the average contact number of any two regions which have the same genomic distance as the two regions. For the networks of gene and transcription factor binding sites, we set a sequence-depth-dependent threshold to filter out interactions with low contact numbers so that the same type of networks of all the four cell/cell lines have the same number of edges (i.e. interactions).

### Generating intra- and inter-chromosomal contact matrices

Intra-chromosomal and inter-chromosomal contact matrices were generated by counting numbers of mapped read contacts falling into each pair of equal-length regions/segments of chromosome(s). The length of the segment (i.e. the resolution of a contact matrix) can be adjusted according to research goals. We used 1Mb resolution for comparing interaction patterns between different cell lines, but used much smaller resolutions, 0.1 Mb and 0.01 Mb, in order to identify the boundaries of chromosomal translocations. Similarly as in [13], the number of short-range contacts is much larger than long-range ones. In order to make the long-range contacts easy to observe in the visualized heat maps of contact matrices, we set a maximum cap (e.g. 50) on the number of contacts between two regions in contact matrices for visualization.

We normalized the chromosomal contact matrices in order to (1) discover the statistically enriched and depleted regions within one contact matrix and (2) compare two contact matrices to recognize the differences in their interaction patterns. For example, by comparing the normalized inter-chromosomal contact matrices of the healthy and Leukemia cell samples, we discovered the translocation between chromosome 11 and 14 in the Leukemia cell line. A variety of normalization methods were tested, including *x/avg*, *(x-min)/max*, and *(x-mean)/sd*, where *x*, *max*, *min*, *mean*, and *sd* are the number of contacts between two regions *i* and *j*, maximum, minimum, mean, and standard deviation of contact numbers in the matrix, respectively. The intra-chromosomal heat maps of the Primary ALL, MHH-CALL-4, RL, and healthy B cell lines can be found at **Fig. S5, S6, S7, S8, S9, S10, S11, and S12**. The heat maps were generated using the “heatmap.2” package in R.

### Statistical significance analysis of chromosomal contact matrix

To infer the statistical significance of the number of contacts, we assumed that contact values excluding local contacts on the diagonal line in a contact matrix follow a Poisson distribution. Each value in the statistical significance matrices is the probability of observing a higher contact number for a pair of locations by chance. The smaller probability value in the significance matrix indicates the higher statistical significance. **Fig. S14** shows the significance matrices of the three malignant cell samples.

### Construction of Pearson’s correlation matrix from chromosomal contact matrix

The correlation matrices were generated based on the contact matrices mentioned above. The value *C_i, j_* of a cell in a correlation matrix is the Pearson correlation between the values in the *i*th and *j*th rows in the contact matrix containing absolute contact numbers between region *i* and *j*. If the *i*th and *j*th regions are in contact, they likely share similar contact partners, leading to a higher correlation. The Pearson’s correlation matrices can effectively reduce the noise in contact data to amplify the plaid contact patterns corresponding to open and closed chromatin conformations. The contact correlation matrices were also used to discern the genome conformation difference between different cell lines. The difference matrix between two correlation matrices *C*_1_ and *C*_2_ equals to |*C*_1_ – *C*_2_|, which can be visualized as heat maps to show differences in chromosomal conformations.

### Calculation of observed/expected numbers of contacts between all pairs of chromosomes

We generated the observed/expected number of inter-chromosomal contacts between each pair of chromosomes for all four cell lines. The contact numbers between 23 pairs of chromosomes provide a global view that can be used to distinguish conservation and variation in genome conformation between different cell samples as shown in **Figure** **3**. As in [13] , the expected number of contacts between chromosome *i* and *j* was calculated by:
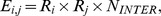
where 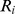 and 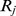 are the fractions of inter-chromosomal reads associated with *i* and *j*, respectively, and 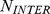 is the total number of inter-chromosomal reads for a cell sample. The actual observed number of inter-chromosomal contacts between chromosomes *i* and *j* divided by the expected number 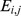 indicates the enrichment or depletion of inter-chromosomal contacts between them.

### Construction of gene-gene interaction networks from Hi-C chromosomal contact data

The gene definitions of the human genome (build 36.3) were downloaded from the NCBI website. We only kept the “GENE” entries excluding the other types including “PSEUDO”, “RNA”, “CDS”, and “UTR”. A gene is denoted by a node/vertex in the gene-gene interaction network that represents all the spatial gene-gene interactions in a chromosome or a genome. An edge between two nodes is added if the number of Hi-C contacts between the two genes is higher than the contact threshold. Different contact thresholds are chosen for each of the four cell lines’ gene-gene interaction networks in order to make them have equal number of edges. **Figure** **4** shows the intra-chromosomal gene-gene interaction network and its properties (node degree distributions, shortest path length distribution, average clustering coefficient distribution, closeness centrality, stress distribution, and topological coefficients) for the chromosome 14 of the CALL-4 cell line. The degree of a node is the number of edges linked to it. The node degree distribution depicts the number of nodes having various degree values. The shortest path length distribution indicates the number of node pairs who have a shortest path *k* between them, *k* = *1*, *2*, *3*…. The clustering coefficient of a node *n* is calculated by:
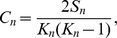
where 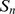 is the number of edges among immediate (one edge away) neighbors of node *n*, and 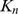 is the number of immediate neighbors of the node *n*. Clustering coefficient is between 0 and 1 indicating the tendency of immediate neighboring nodes to be clustered. Average clustering coefficient is calculated by averaging the clustering coefficients of all nodes in the network.

The closeness centrality 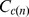 of a node *n* is calculated as:
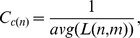
where 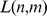 is the length of the shortest path between the nodes *n* and any other node *m*. Closeness centrality of a node is between 0 and 1 measuring how fast information can be broadcasted from one node to other reachable nodes. Isolated nodes having a closeness centrality 0 were not considered in **Figure** **4**. The stress of a node *n* is the number of shortest paths traversing through node *n*; and the stress distribution indicates the number of nodes with specific stress values. The stress values are grouped into bins whose sizes are factor of 10, i.e., 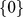, 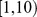, 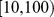
…. The topological coefficient 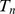 of a node *n* is calculated as:



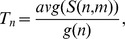



where *m* is a node that shares at least one neighbor with node *n* or there is a direct link between node *m* and node *n*, function 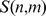 returns the number of neighbors shared between node *m* and node *n*, with 1 added if there is a direct link between node *m* and node *n*, and 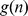 is the number of immediate neighbors node *n*. The topological coefficient of a node measures the extent to which a node shares neighbors with other nodes. The topological coefficient of a node having zero or one neighbor is assigned to 0.

### Construction of interaction network of transcription factor binding sites (TFBS)

The definitions and coordinates of transcription factor binding sites were downloaded from Yale TFBS [31], which were identified by ChiP-seq experiments. In the TFBS networks, a node denotes a TFBS. Two TFBS nodes are connected by an edge if the Hi-C contacts between them is higher than the contact threshold. The sequence-depth-dependent contact threshold is set to make the number of edges of networks of all four cell/cell lines equal. The weight of the edge is the number of Hi-C contacts between the two nodes. We generated a genome-wide TFBS-TFBS interaction network including both intra- and inter-chromosomal contacts of all 23 pairs of chromosomes.

### Construction of interaction networks of transcription factor binding sites (TFBS) and genes

Based on the definitions of TFBS downloaded from Yale TFBS [31] and the NCBI gene definitions, we constructed the TFBS-gene interaction networks from the Hi-C contact data using the same approach described above.

## Supporting Information

Figure S1
**The distribution of the sequencing qualities (Solexa-scale) of paired-end reads of the two malignant primary ALL B-cell data sets (i.e. quality scores V.S. nucleotide positions).** The sequencing quality score at a position is calculated as 
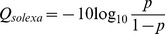
, where *p* is the probability of a sequencing error at the position. A score 30 means the probability of a sequencing error at the position is ∼ 0.001. A score 20 or above may be considered acceptable. The plots show the median (the black curve), 1^st^ and 91^st^ percentiles, 2^nd^ and 3^rd^ quartiles from positions 1 to 120 in the reads data.(JPG)

Figure S2 **The distribution of the sequencing qualities of paired-end reads of the two malignant MHH-CALL-4 cell line data sets.**
(JPG)

Figure S3 **The distribution of the sequencing qualities of paired-end reads of the two malignant lymphoma RL cell line data sets.**
(JPG)

Figure S4 **The plots of contact numbers against regions of chromosome 7, 11 and 14 of four cell samples and the plots of gene numbers against regions of chromosome 7, 11 and 14.** The X-axis in Plots A-L denotes chromosomal region index at resolution 1Mb and the Y-axis denotes the number of intra- and inter-chromosomal contacts in each region. An inter-chromosomal contact is a spatial contact between two different chromosomes, and an intra-chromosomal contact a contact within the same chromosome. A, B and C are the plots of chromosomes 7, 11, and 14 for the MHH-CALL-4 cell line respectively, D, E and F for the RL cell line, G, H and I for the normal B-Cell, and J, K and L for the Primary B-ALL cell. The plots show that the number of contacts generated from the sequence data is not evenly distributed along the chromosomes. The extra M, N and O plots show the number of genes in each region against the regions of chromosome 7, 11 and 14 separately.
(JPG)

Figure S5 **The intra-chromosomal contact heat maps for all chromosomes of the primary ALL B-cell.** Interested readers may contact us for images with higher resolution and for contact matrix data.
(JPG)

Figure S6 **The intra-chromosomal contact heat maps normalized by using SCN procedure for all chromosomes of the primary ALL B-cell.** Interested readers may contact us for images with higher resolution and for contact matrix data.
(JPG)

Figure S7 **The intra-chromosomal contact heat maps for all chromosomes of the MHH-CALL-4 cell line.** Interested readers may contact us for images with higher resolution and for contact matrix data.
(JPG)

Figure S8 **The intra-chromosomal contact heat maps normalized by using SCN procedure for all chromosomes of the MHH-CALL-4 cell line.** Interested readers may contact us for images with higher resolution and for contact matrix data.
(JPG)

Figure S9 **The intra-chromosomal contact heat maps for all chromosomes of the RL cell line.** Interested readers may contact us for images with higher resolution and for contact matrix data.
(JPG)

Figure S10 **The intra-chromosomal contact heat maps by using SCN procedure for all chromosomes of the RL cell line.** Interested readers may contact us for images with higher resolution and for contact matrix data.
(JPG)

Figure S11 **The intra-chromosomal contact heat maps for all chromosomes for the normal B-cell line.** Sequence reads data were downloaded from Lieberman-Aiden etc [13]. Mapping and construction of contact maps were carried out by our pipeline.
(JPG)

Figure S12 **The intra-chromosomal contact heat maps by using SCN procedure for all chromosomes for the normal B-cell line.** Sequence reads data were downloaded from Lieberman-Aiden etc [13]. Mapping and construction of contact maps were carried out by our pipeline.
(JPG)

Figure S13 **The Pearson’s correlation matrix for intra-chromosomal contact numbers between the normal B cell, primary ALL B-cell, MHH-CALL-4 cell line, and RL cell line.** For each cell, the number of intra-chromosomal contacts for each of 23 pairs of chromosomes was calculated and was put into a vector. Thus, each cell sample has one intra-chromosomal contact vector. The matrix below shows the Pearson’s correlation between each pairs of vectors of two cell samples.
(JPG)

Figure S14 **Contact significance analysis of selected chromosomes.** In order to check if the number of contacts between two specific chromosome regions is significantly large, we calculated the significance score (i.e. the probability of receiving this number of contacts or more) in each cell of an intra-chromosome contact matrix at 1Mb resolution, assuming the background distribution of contact numbers follows the Poisson distribution. The parameter (lamda: mean contact number) of the background distribution was set to the average of number of contacts in the matrix excluding contacts within the same region (i.e. diagonal line in a matrix). Sub-figures A, B, C and D illustrate the contact significant scores of the intra-chromosomal contract matrices of chromosome 7 of the MHH-CALL-4 cell line, RL cell line, normal B-cell and the Primary B-ALL cell, respectively. Sub-figures E, F, G and H depict the significance scores of intra-chromosomal contact matrices of chromosome 14 of the MHH-CALL-4 cell line, RL-cell line, normal B-cell line and the primary B-ALL cell, respectively. Darker red indicates higher significant score.
(JPG)

Figure S15 **The inter-chromosome contact matrix between chromosome 11 and 14 of Normal B-Cell Line.**
(JPG)

Figure S16 **The method of calculating the corrected inter-chromosomal contact matrix of translocated chromosomes.** (**A**) Division of the corrected inter-chromosomal contact matrix between chromosomes 11 and 14 into three regions to be reconstructed separately. Region A contains the contacts between non-translocated segments in chromosome 11 (i.e. 11(c) and 11(d) in (**B**)) and non-translocated segments in chromosome 14 (i.e. 14(c) and 14(d) in (**B**)). Region B contains the contacts between translocated segments in chromosome 11 (i.e. 11(b) in (**B**)) and translocated segments in chromosome 14 (i.e. 14(a) in (**B**)). Region C contains the contacts between non-translocated segments in chromosome 11 (i.e. 11(c) and 11(d) in (**B**)) and translocated segments in chromosome 14 (i.e. 14(a) in (**B**)). Region D contains the contacts between non-translocated segments in chromosome 14 and translocation segment in chromosome 11. For the contacts in regions A and B, we divided the original contact numbers by 2 in order to estimate the inter-chromosome contacts. For region C, we normalized the value of each cell C_ij_  =  max (0, C_ij_ – average num of row i in region A). For region D, we normalized the value of each cell D_ij_  =  max(0, D_ij_ – average num of column j in region A).
(JPG)

Figure S17 **The corrected inter-chromosomal contact map between translocated chromosomes 11 and 14 for the primary ALL B-cell.** The method of calculating it can be found in Figure S13.
(JPG)

Figure S18 **The Pearson’s correlation matrix for inter-chromosomal contact numbers between the normal B cell, primary ALL B-cell, MHH-CALL-4 cell line, and RL cell line.** For each cell, the number of inter-chromosomal contacts between chromosomes were calculated and put into a vector. Thus, each cell has one vector to represent all its inter-chromosomal contact numbers. The matrix below shows the Pearson’s correlation between each pairs of vectors of two cell samples.
(JPG)

Figure S19 **The distribution of inter and intra chromosome contact number for Normal-B Cell before using SCN procedure and after using SCN procedure.** The number of inter contact after using SCN is calculated by summing up all inter chromosome contact matrix normalized by using SCN procedure, and then divided by the number of inter chromosome contact matrix. The number of intra contact after using SCN is calculated by summing up all intra chromosome contact matrix normalized by using SCN procedure, and then divided by the number of intra chromosome contact matrix.
(JPG)

Figure S20 **The number of contacts between 13 genes in the cluster in each cell line.**
(JPG)

Figure S21 **The visualization of reads mapped to the HoxA gene region (27,104,502 – 27,212,501) on chromosome 7 of the human genome by the UCSC genome browser.** The vertical line segments under the label “chromosome contact” denote the locations where the reads were mapped to. The reads data of the MHH-CALL-4 cell line was used.
(JPG)

Figure S22 **The networks of inter-chromosomal gene-gene interactions between chromosome 11 and 14 for RL-Cell Line.**
(JPG)

Figure S23 **Node-degree distribution of Figure 22.**
(JPG)

Figure S24 **Shortest path frequency of Figure 22.**
(JPG)

Figure S25 **The distribution of topological coefficients of the networks shown in Figure 22.**
(JPG)

Figure S26 **The interaction network between transcription factor binding sites (TBSs) in the entire genome of the MHH-CALL-4 cell line.** This is generated based on raw contacts.
(JPG)

Figure S27 **The distribution of node degree of the TBS-TBS interaction network shown in Figure S26.**
(JPG)

Figure S28 **The histogram of lengths of the shortest paths between any two nodes in the TBS-TBS interaction network shown in Figure S26.**
(JPG)

Figure S29 **The distribution of topological coefficients the TBS-TBS interaction network shown in Figure S26.**
(JPG)

Figure S30 **The distribution of node stresses of the TBS-TBS interaction network shown in Figure S25.**
(JPG)

Figure S31 **The interaction network between transcription factor binding sites (TBSs) of chromosome 4 and chromosome 4 of the MHH-CALL-4 cell line.** This network is normalized with the contact threshold 2.
(JPG)

Figure S32 **The distribution of node degree of the TBS-TBS interaction network shown in Figure S31.**
(JPG)

Figure S33 **The histogram of lengths of the shortest paths between any two nodes in the TBS-TBS interaction network shown in Figure S31.**
(JPG)

Figure S34 **The spatial interaction networks between genes and transcription factor binding sites (TFB) in chromosome 14 for the CALL-4 cell line.** A node in the network denotes a gene or a TFB. Two nodes are connected by an edge if they are spatially contacted.
(JPG)

Figure S35 **The node degree distribution of the network shown in Figure S34.** It is shown that the frequency (number of nodes) is largely linear to the degree of the nodes on the log-log scale. This suggests that the network is likely a scale-free network.
(JPG)

Figure S36 **The histogram of lengths of the shortest path between any two nodes in the network shown in Figure S34.**
(JPG)

Figure S37 **The distribution of stress values of the network shown in Figure S34.**
(JPG)

Figure S38 **The distribution of topological coefficients of the network shown in Figure S34.**
(JPG)

Figure S39 (**A**) The chromosomal region of the transcription factor binding site on chromosome 14 of the MHH-CALL-4 cell line that has the highest contacts with other genes is visualized by the UCSC genome browser. This transcription factor binding site contacted 1460 times with GeneID:145508 (starting from the position 61002767 and ending at 61445813), 1 time with GeneID:7253, 2 times with GeneID:6710, 2 times with GeneID:56659, and 1 time with GeneID:9369. (**B**) The chromosomal region of the gene (GeneID:145508) that encodes a centrosomal protein (128 kDa). More information about this gene is available at http://www.ncbi.nlm.nih.gov/sites/entrez?db=gene&term=145508.
(JPG)

Figure S40 **Ligation efficiency.** Both the 3C and Hi-C libraries should run as a fairly tight band larger than 10 Kb. Ligation efficiency is slightly lower in Hi-C than in 3C and is indicated by the smear in the Hi-C lanes (see van Berkum et al. in JoVE for a complete description). The right triangles above each panel represent increasing or decreasing amounts of template. The Lambda HindIII ladder (λ) and a 1 Kb DNA ladder were also included on the visualization gels. The uppermost fragment of the lambda HindIII ladder is 23.13 Kb and the uppermost fragment of the 1 Kb ladder is 10 Kb. A quantative agarose gel was run (0.8%) on an acute lymphoblastic leukemia cell line (A), an acute lymphoblastic leukemia patient sample (B) and a follicular lymphoma cell line (C). Panel C includes 5 Hi-C template amounts and one 3C template amount whereas panels A and B both include 2 Hi-C and 2 3C template amounts.
(JPG)

Figure S41 **PCR digest control.** A quantative agarose gel was run (0.8%) on an acute lymphoblastic leukemia cell line (A), an acute lymphoblastic leukemia patient sample (B) and a follicular lymphoma cell line (C). During Hi-C the HindIII site is lost and an NheI site is created (see van Berkum et al. in JoVE for a complete description of the protocol) and the products can be distinguished from a 3C experiment by digesting the ligation site. The digested samples were quantified using ImageJ software. A total of three PCR reactions were done for both 3C and Hi-C samples. The reactions were pooled and purified using the Zymo clean and concentrator kit per the manufacturer’s protocol eluting 2x with 10 µL with water. The PCR products were digested with HindIII (no blunting of the DNA ends) or NheI (shows blunting and biotin incorporation). 56% (FL Cell Line), 64% (ALL Cell Line) and 68% (Patient Sample) of Hi-C amplicons were digested by NheI confirming the efficient marking of ligation junctions.
(JPG)

Figure S42 **An overview of the bioinformatics pipeline of analyzing Hi-C experimental reads data.** The Hi-C wet lab experiment is similar to the method described in [13], in which chromosome DNA is cross-linked, ligated and then sheared. Each of the reads-pairs was mapped to the human genome by the tool maq (http://maq.sourceforge.net/) with the mistake threshold (–e) set to 150. Our computer programs analyzed the mapping output and handled the four different cases in which the reads may cover different portions of the two chromosomes. These four situations are illustrated in the following figure. Case one is that each of the two ends can only be mapped to one location and the two mapped locations of the two ends are 2000b away. Case two is one end can be mapped to one location (e.g. location A), but the other end to two locations (e.g. B and C). In this case, we checked whether one of the two locations (B or C) is within 2000b of A. If not, the case is considered invalid and is discarded. 2000bp was used as the threshold because the average length of the DNA insert is 2000bp long. Case three is the same as Case two except that the first end was mapped to two locations and the second to one location. Case four is both two ends can be mapped to two locations (e.g. one end to A and B, and the other to C and D as shown in the figure. A, B, C, and D are the starting positions of the mapping locations). In this case, we checked whether the distance between A and C is less than the read length and whether the distance between B and D is less than the read length. If yes, they were kept. In our first mapping strategy, only these four cases above were considered and processed to generate contacts and all the other cases were discarded. For example, if one end of a pair of ends can be mapped to > = 3 locations, they were discarded. This process was able to reduce noise (e.g. wrongly-aligned reads) and ensure the quality of contact parsing. We also developed the second simplified strategy to control mapping quality and minimize mapping ambiguities. That is, only keep the reads that can be uniquely mapped to one location of the chromosome. If one of the two ends was mapped to > = 2 locations, these pair of ends were discarded. We found these two strategies generated similar contacts. The results presented in this paper were based on contacts generated by the second strategy that is more stringent. The intra- and inter-chromosomal contact matrices for chromosomes and chromosome pairs were visualized as heat maps by the statistical package R.
(JPG)

Figure S43 **The distribution of Euclidean Norm of each region of both inter and intra chromosome contact for MHH-CALL-4.** Figure S37(A) shows the distribution of Euclidean Norm of each region of chromosome 14 which has inter chromosome contact with chromosome 20 of MHH-CALL-4, we can see a Gaussian distribution for the Euclidean Norm, and we set 10 as a threshold, all regions which have Euclidean Norm less than 10 will be removed. The resolution for each region is 1M. Figure S37 (B) shows the distribution of Euclidean Norm of the intra chromosome contact of chromosome 14 of MHH-CALL-4. The resolution for each region is 10M. We set 10 as the threshold to normalize the contact map. The threshold 100 is set to normalize the intra contact map when the resolution for each region is 1M.
(JPG)

Table S1 **Reads coverage of the gene regions and non-gene regions.** The reads coverage of gene region was calculated as the reads length multiply the number of contact in gene region/total length of gene region. The coverage of non-gene region was calculated as the read length * number of contact not in gene region/total length of non-gene region. Here the gene length was calculated according to the gene start and end information.
(DOCX)

Table S2 **Total number of reads for all samples mentioned in this work.** The data of the normal B cell was downloaded from the publication [13]. The others were generated by us. One pair-end read pair contains two ends of reads. This table shows the total number of ends. For some cell/cell lines, we sequenced them more than one times and selected the one with the best quality to use in this work.
(DOCX)

Text S1 **The description of materials and methods of the Hi-C experiment.**
(DOCX)
