# Supplementary figures and images for: The Properties of Genome Conformation and Spatial Gene Interaction and Regulation Networks of Normal and Malignant Human Cell Types
Source: PLoS One. 2013 Mar 11;8(3):e58793. doi: 10.1371/journal.pone.0058793 (PMC3594155; doi:10.1371/journal.pone.0058793)

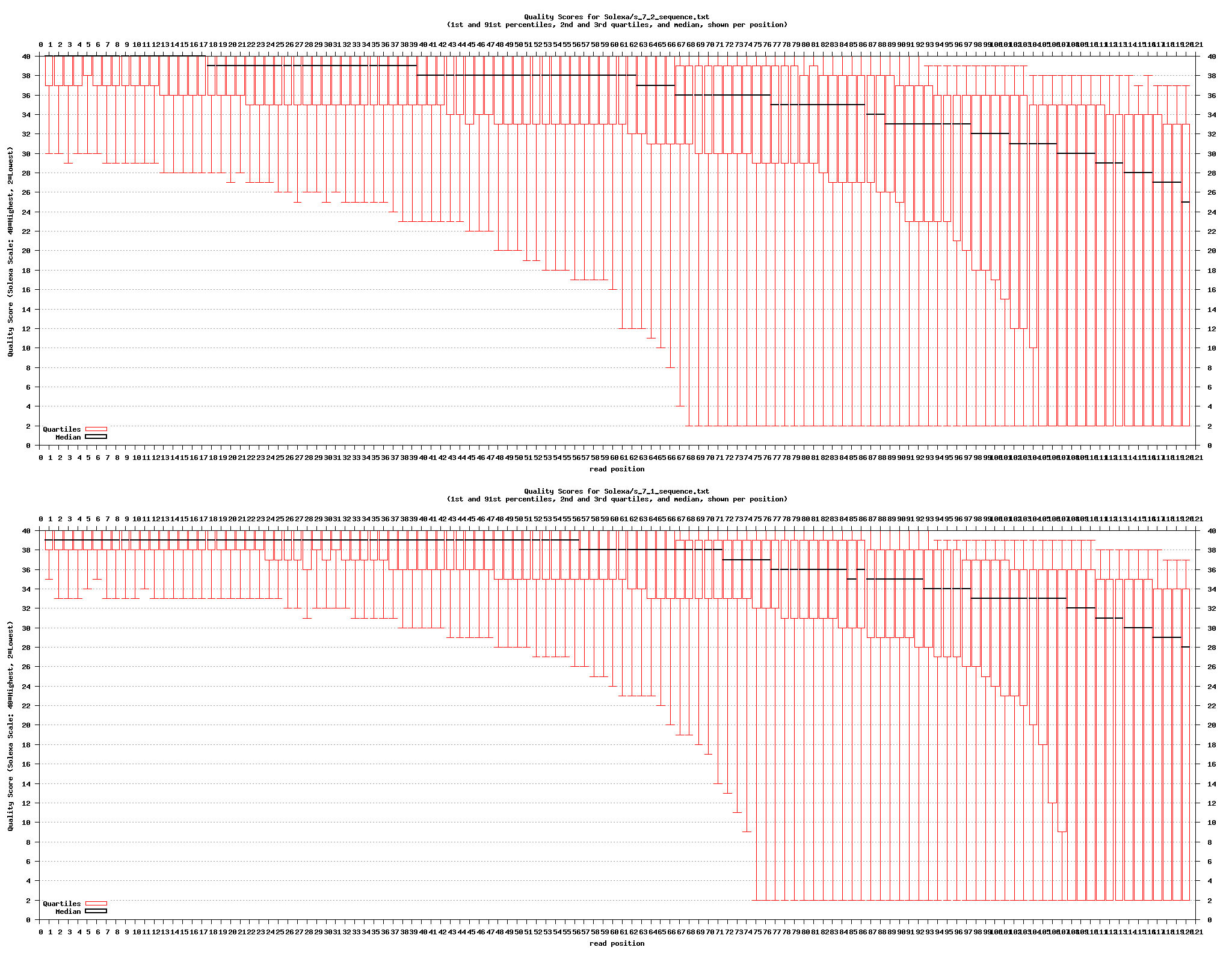

Supplement: Figure S1 — The distribution of the sequencing qualities (Solexa-scale) of paired-end reads of the two malignant primary ALL B-cell data sets (i.e. quality scores V.S. nucleotide positions). The sequencing quality score at a position is calculated as , where p is the probability of a sequencing error at the position. A score 30 means the probability of a sequencing error at the position is ∼ 0.001. A score 20 or above may be considered acceptable. The plots show the median (the black curve), 1st and 91st percentiles, 2nd and 3rd quartiles from positions 1 to 120 in the reads data. (JPG) [file pone.0058793.s001.jpg]

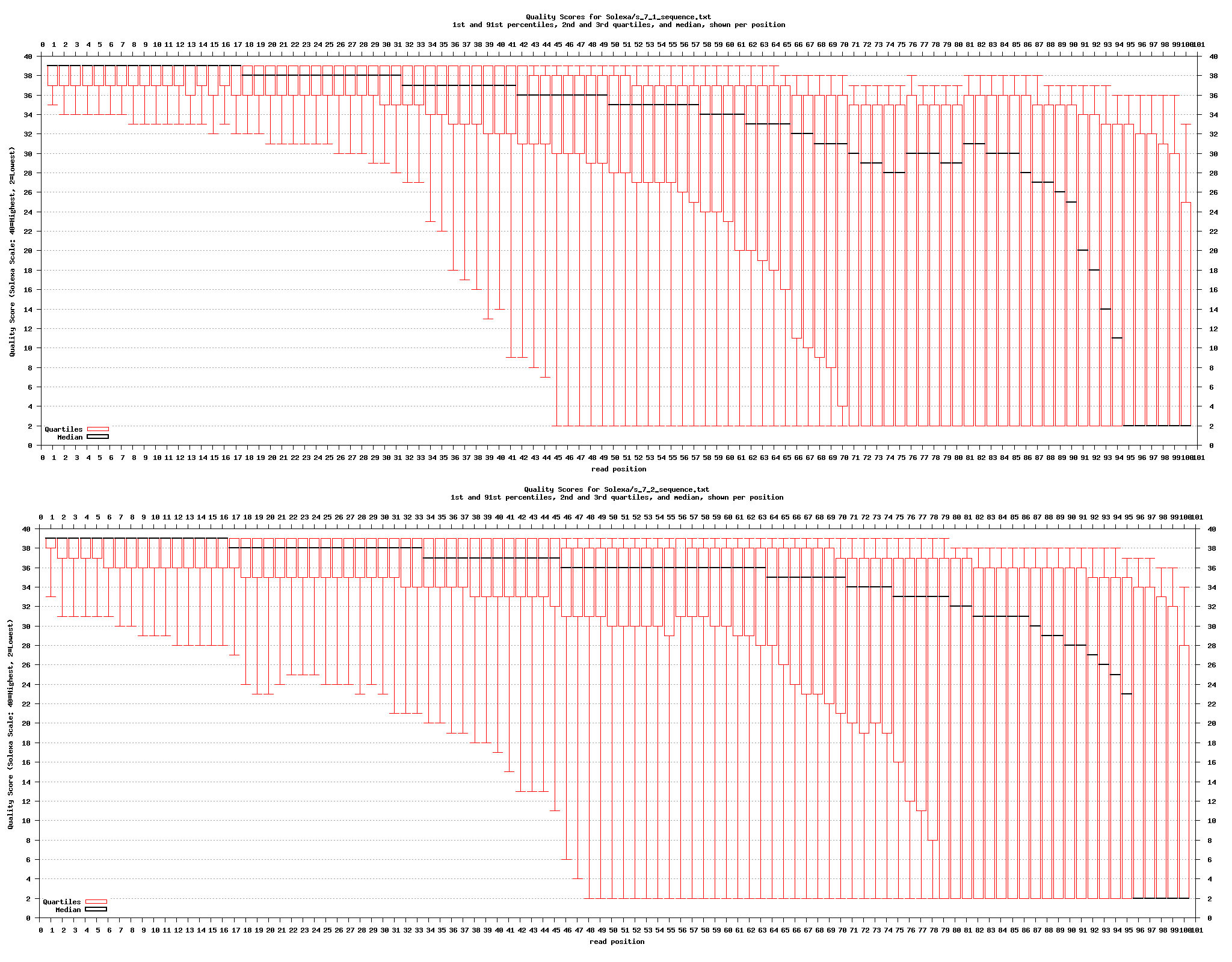

Supplement: Figure S2 — The distribution of the sequencing qualities of paired-end reads of the two malignant MHH-CALL-4 cell line data sets. (JPG) [file pone.0058793.s002.jpg]

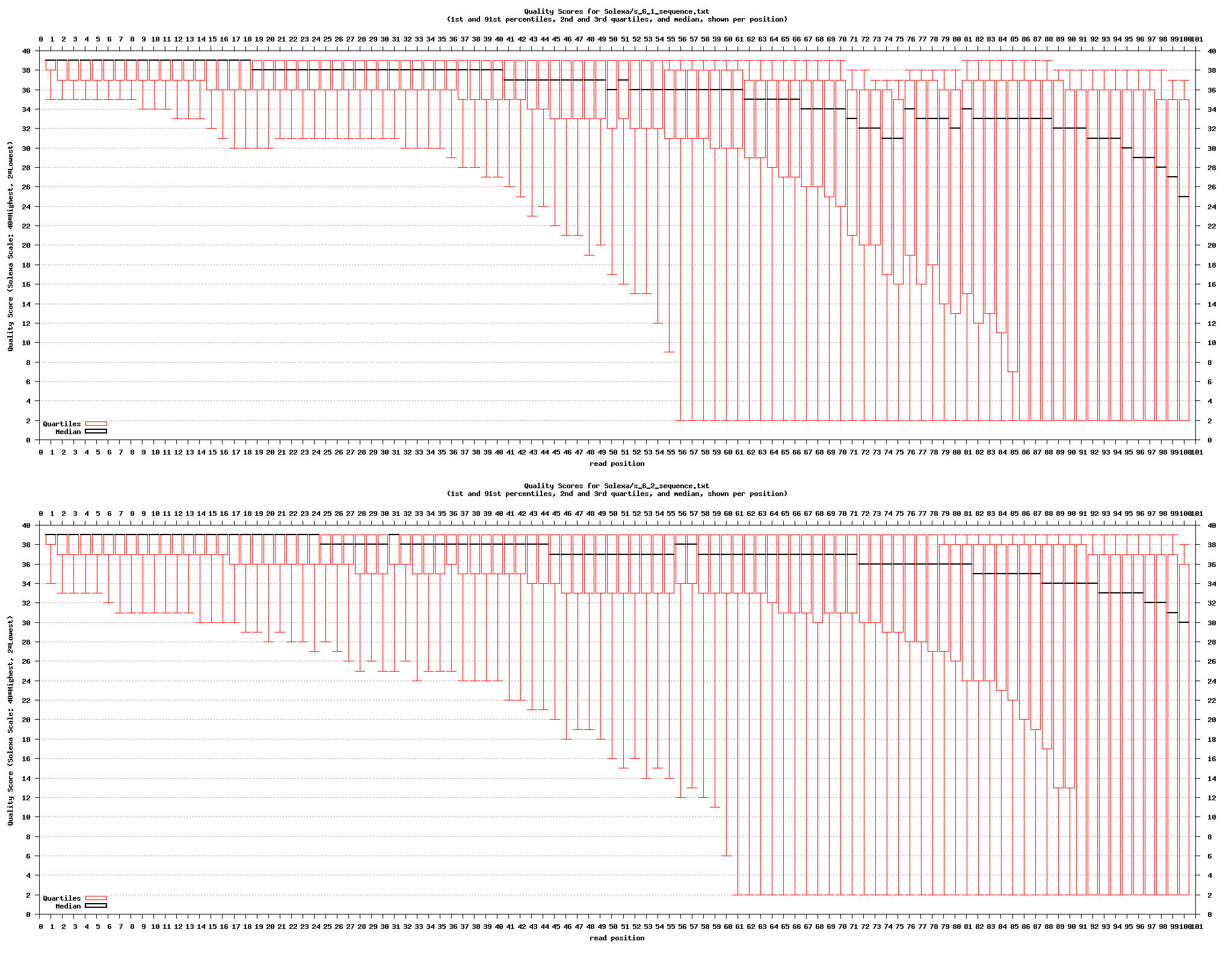

Supplement: Figure S3 — The distribution of the sequencing qualities of paired-end reads of the two malignant lymphoma RL cell line data sets. (JPG) [file pone.0058793.s003.jpg]

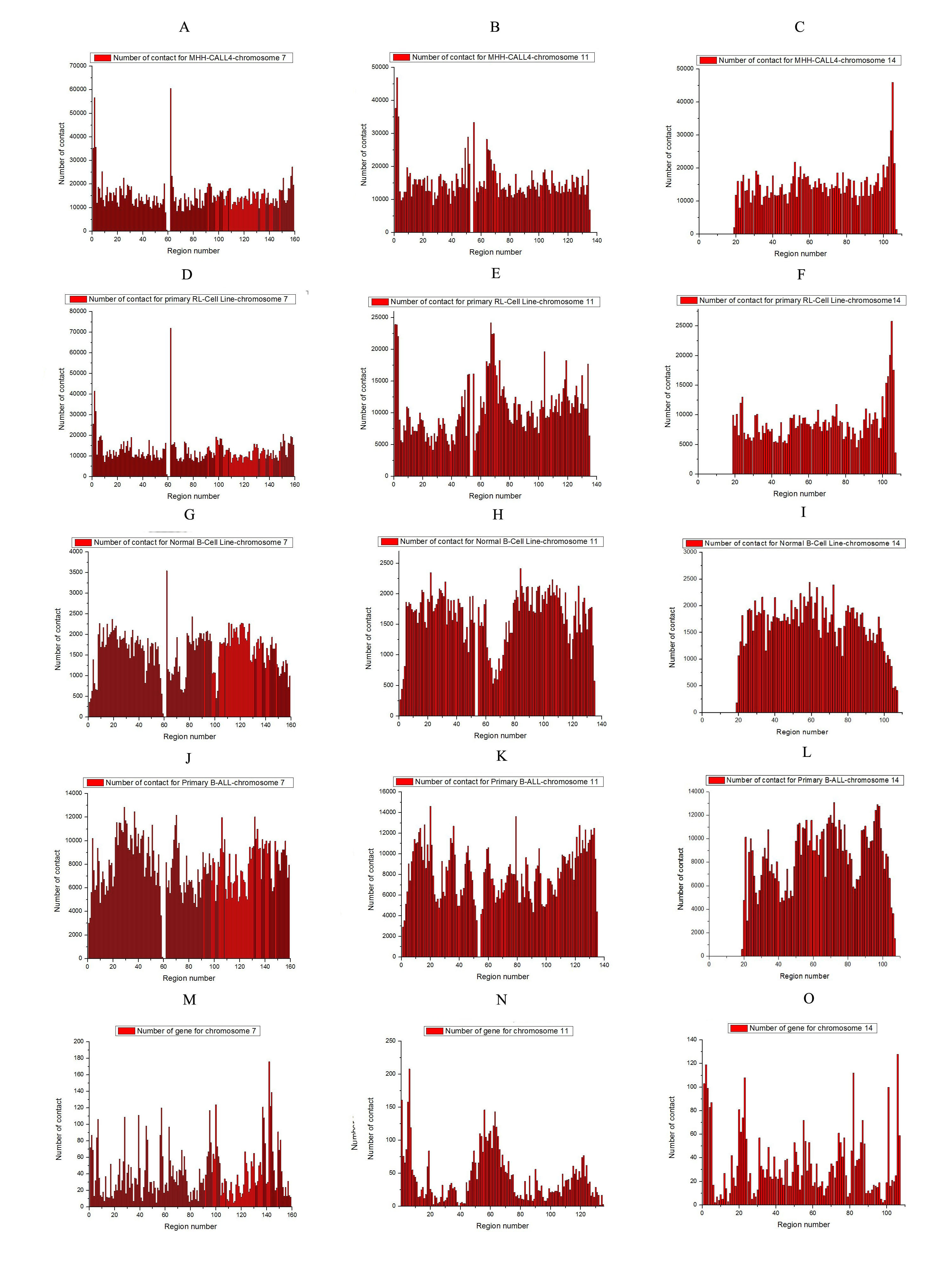

Supplement: Figure S4 — The plots of contact numbers against regions of chromosome 7, 11 and 14 of four cell samples and the plots of gene numbers against regions of chromosome 7, 11 and 14. The X-axis in Plots A-L denotes chromosomal region index at resolution 1Mb and the Y-axis denotes the number of intra- and inter-chromosomal contacts in each region. An inter-chromosomal contact is a spatial contact between two different chromosomes, and an intra-chromosomal contact a contact within the same chromosome. A, B and C are the plots of chromosomes 7, 11, and 14 for the MHH-CALL-4 cell line respectively, D, E and F for the RL cell line, G, H and I for the normal B-Cell, and J, K and L for the Primary B-ALL cell. The plots show that the number of contacts generated from the sequence data is not evenly distributed along the chromosomes. The extra M, N and O plots show the number of genes in each region against the regions of chromosome 7, 11 and 14 separately. (JPG) [file pone.0058793.s004.jpg]

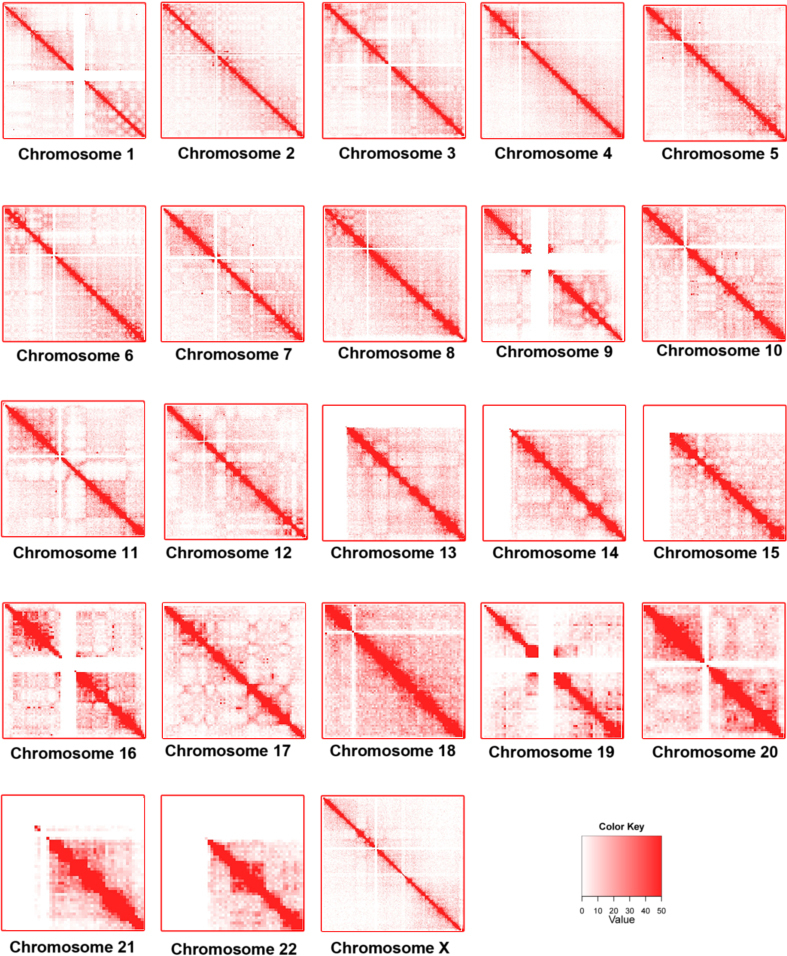

Supplement: Figure S5 — The intra-chromosomal contact heat maps for all chromosomes of the primary ALL B-cell. Interested readers may contact us for images with higher resolution and for contact matrix data. (JPG) [file pone.0058793.s005.jpg]

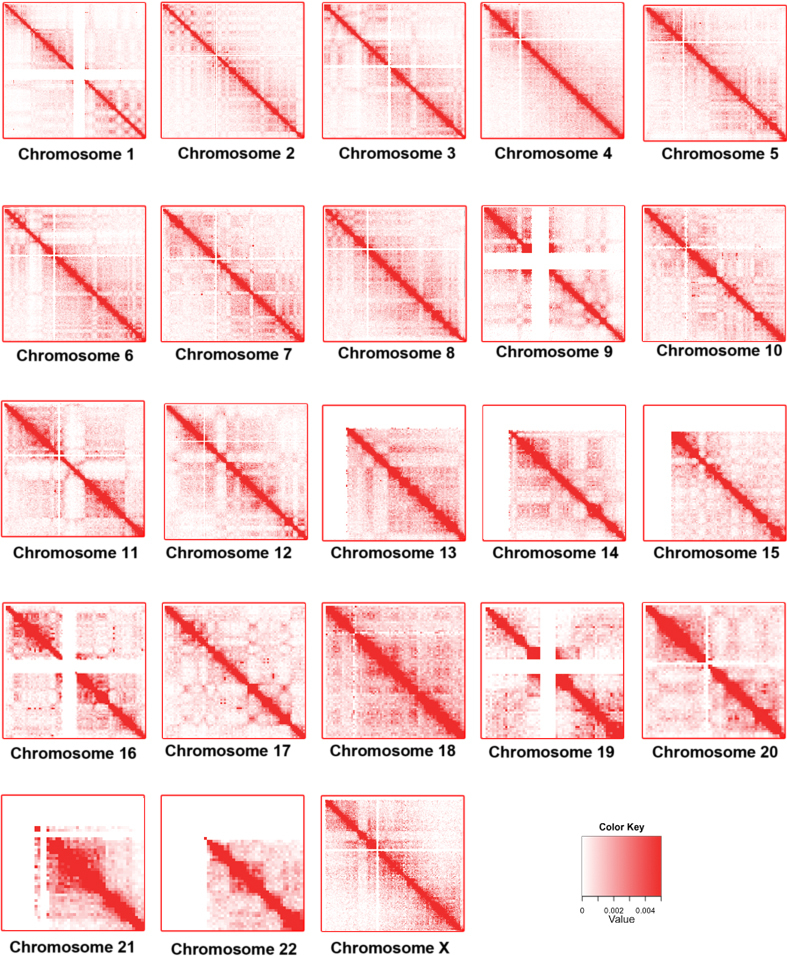

Supplement: Figure S6 — The intra-chromosomal contact heat maps normalized by using SCN procedure for all chromosomes of the primary ALL B-cell. Interested readers may contact us for images with higher resolution and for contact matrix data. (JPG) [file pone.0058793.s006.jpg]

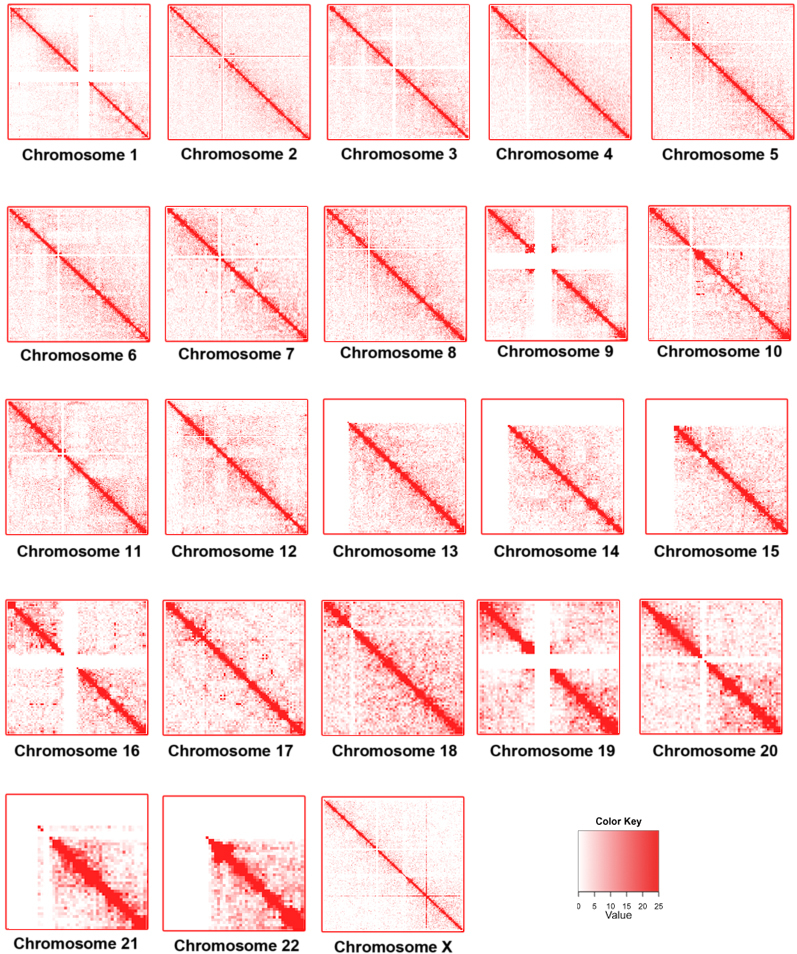

Supplement: Figure S7 — The intra-chromosomal contact heat maps for all chromosomes of the MHH-CALL-4 cell line. Interested readers may contact us for images with higher resolution and for contact matrix data. (JPG) [file pone.0058793.s007.jpg]

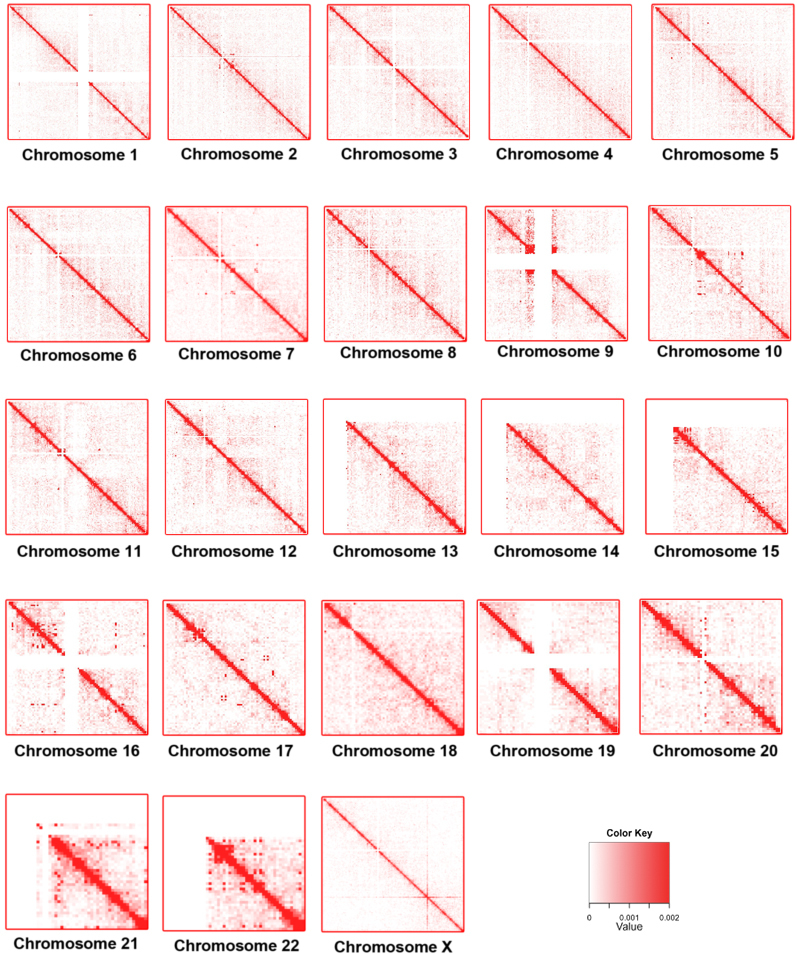

Supplement: Figure S8 — The intra-chromosomal contact heat maps normalized by using SCN procedure for all chromosomes of the MHH-CALL-4 cell line. Interested readers may contact us for images with higher resolution and for contact matrix data. (JPG) [file pone.0058793.s008.jpg]

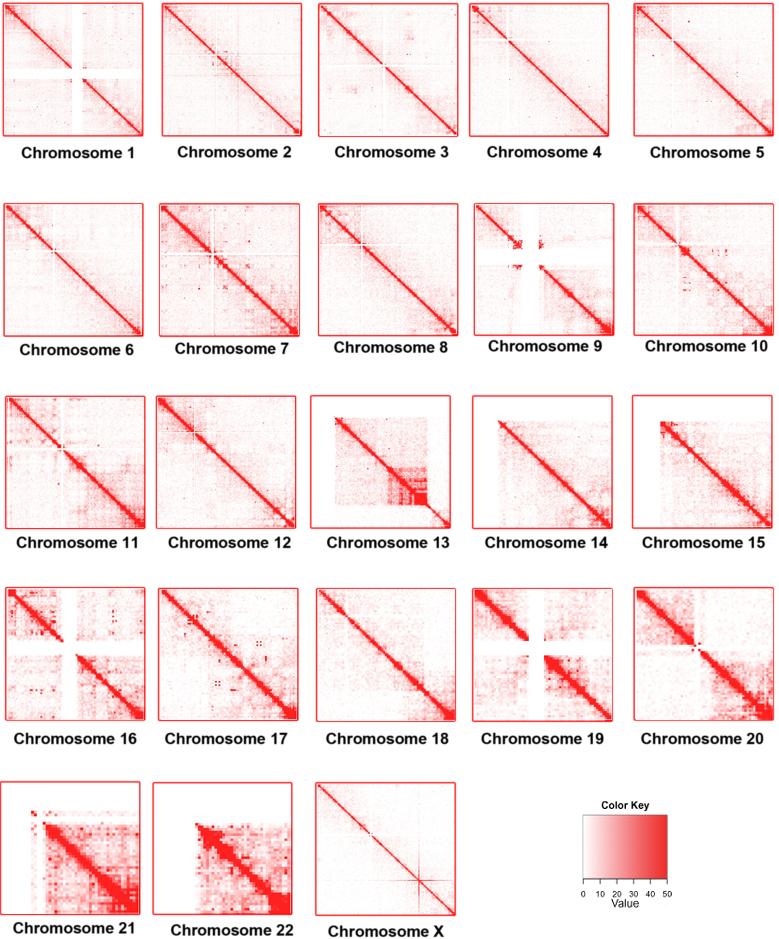

Supplement: Figure S9 — The intra-chromosomal contact heat maps for all chromosomes of the RL cell line. Interested readers may contact us for images with higher resolution and for contact matrix data. (JPG) [file pone.0058793.s009.jpg]

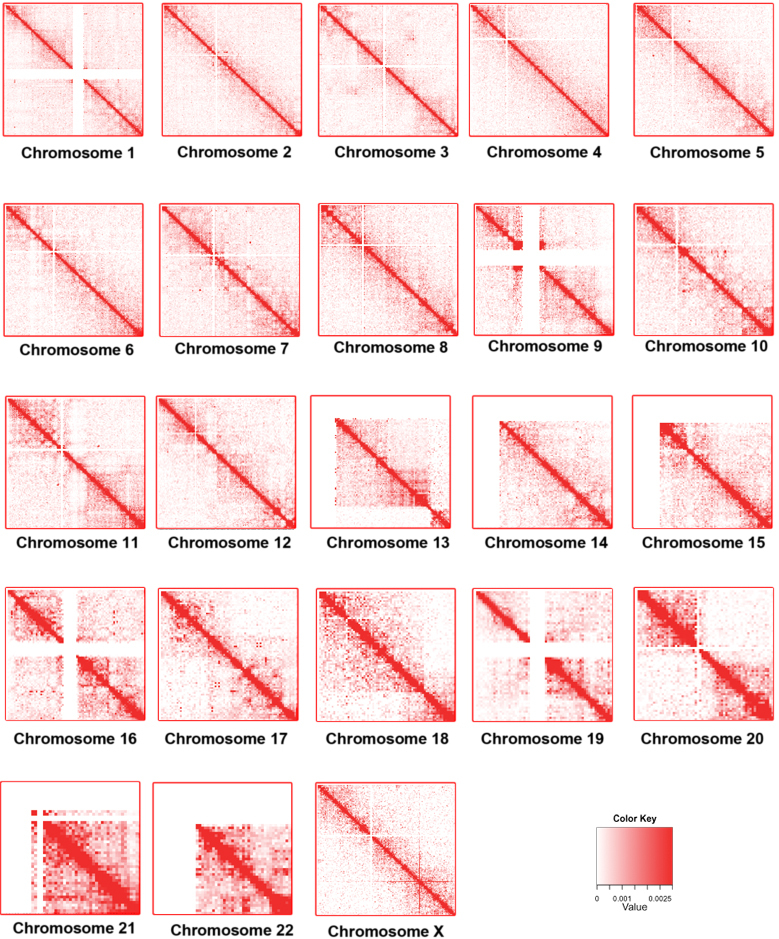

Supplement: Figure S10 — The intra-chromosomal contact heat maps by using SCN procedure for all chromosomes of the RL cell line. Interested readers may contact us for images with higher resolution and for contact matrix data. (JPG) [file pone.0058793.s010.jpg]

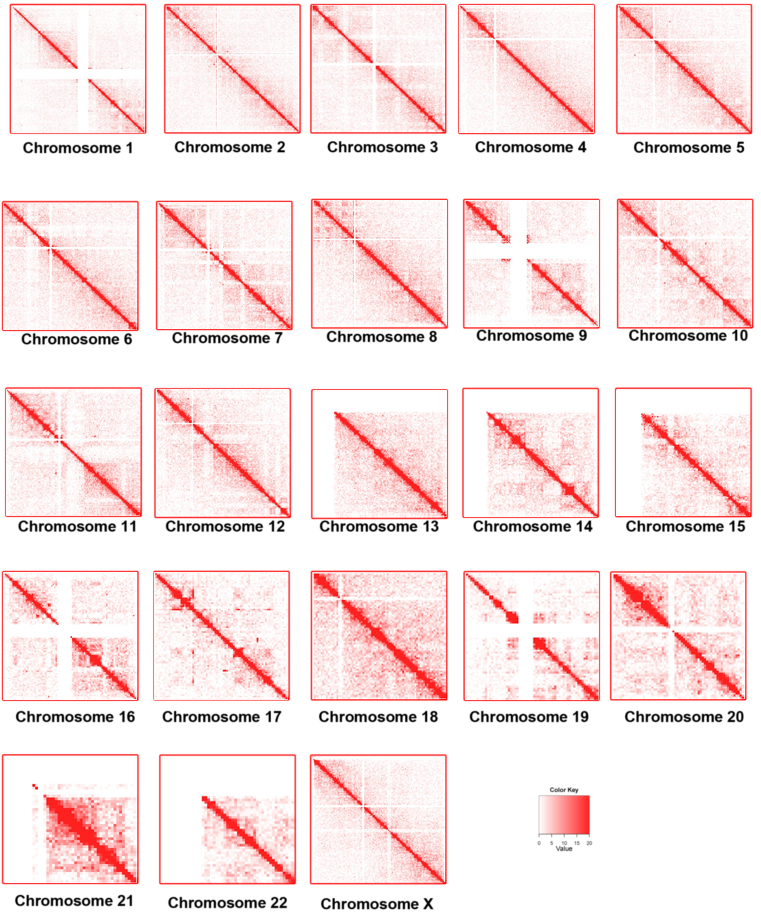

Supplement: Figure S11 — The intra-chromosomal contact heat maps for all chromosomes for the normal B-cell line. Sequence reads data were downloaded from Lieberman-Aiden etc [13]. Mapping and construction of contact maps were carried out by our pipeline. (JPG) [file pone.0058793.s011.jpg]

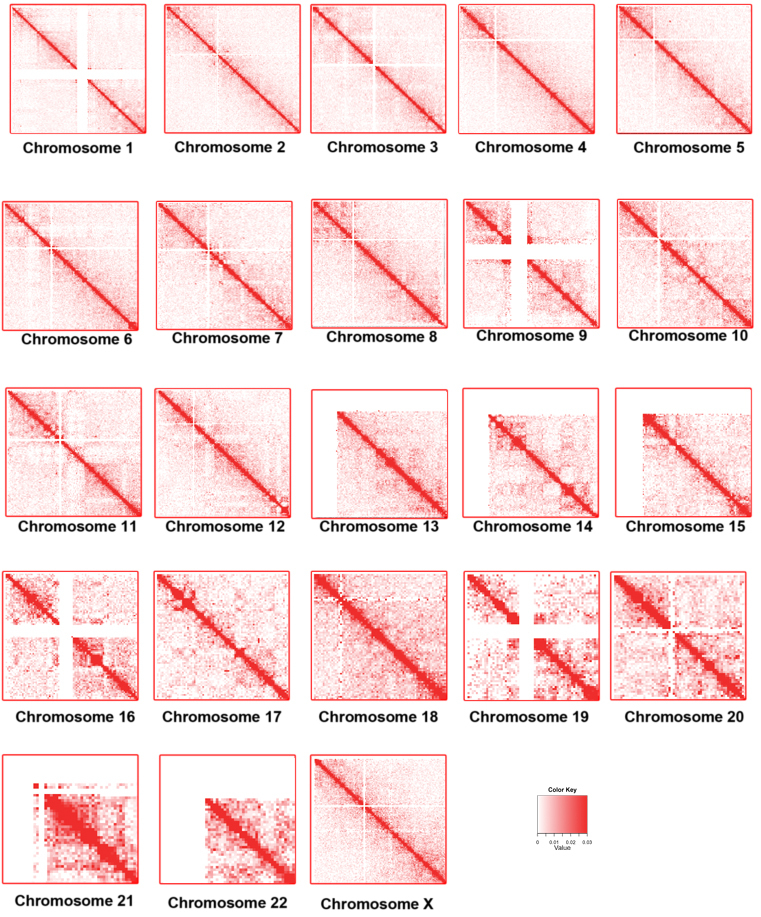

Supplement: Figure S12 — The intra-chromosomal contact heat maps by using SCN procedure for all chromosomes for the normal B-cell line. Sequence reads data were downloaded from Lieberman-Aiden etc [13]. Mapping and construction of contact maps were carried out by our pipeline. (JPG) [file pone.0058793.s012.jpg]

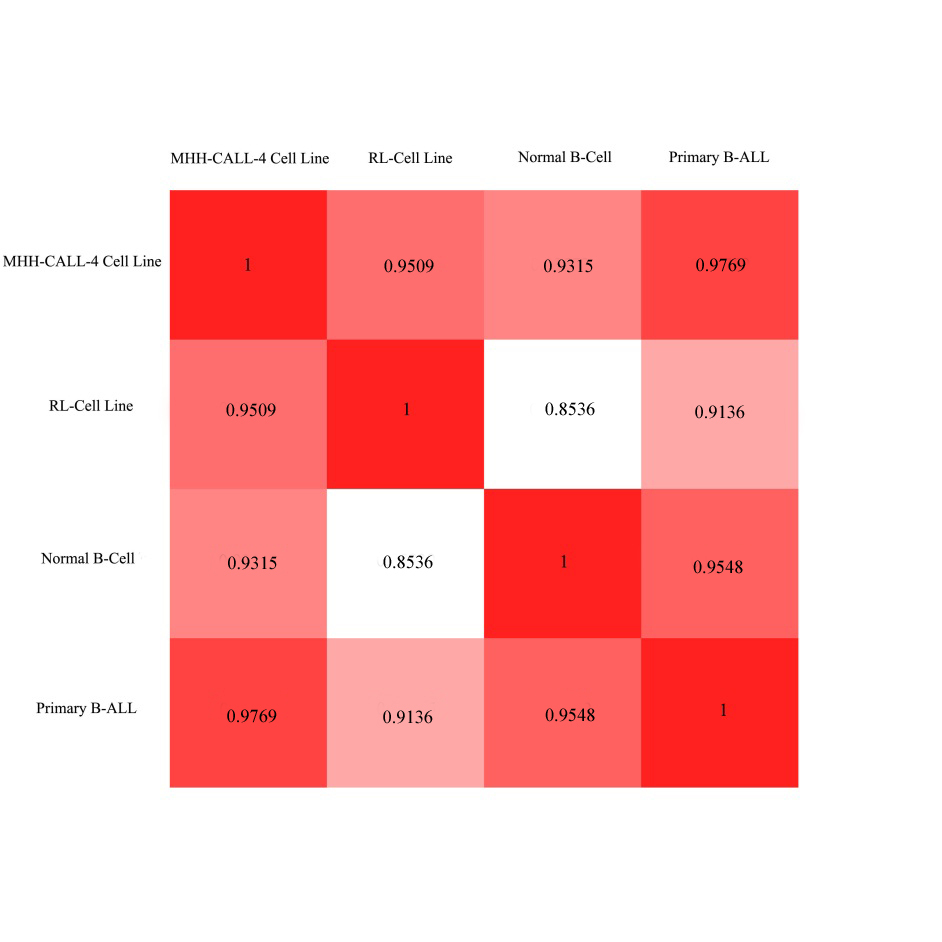

Supplement: Figure S13 — The Pearson’s correlation matrix for intra-chromosomal contact numbers between the normal B cell, primary ALL B-cell, MHH-CALL-4 cell line, and RL cell line. For each cell, the number of intra-chromosomal contacts for each of 23 pairs of chromosomes was calculated and was put into a vector. Thus, each cell sample has one intra-chromosomal contact vector. The matrix below shows the Pearson’s correlation between each pairs of vectors of two cell samples. (JPG) [file pone.0058793.s013.jpg]

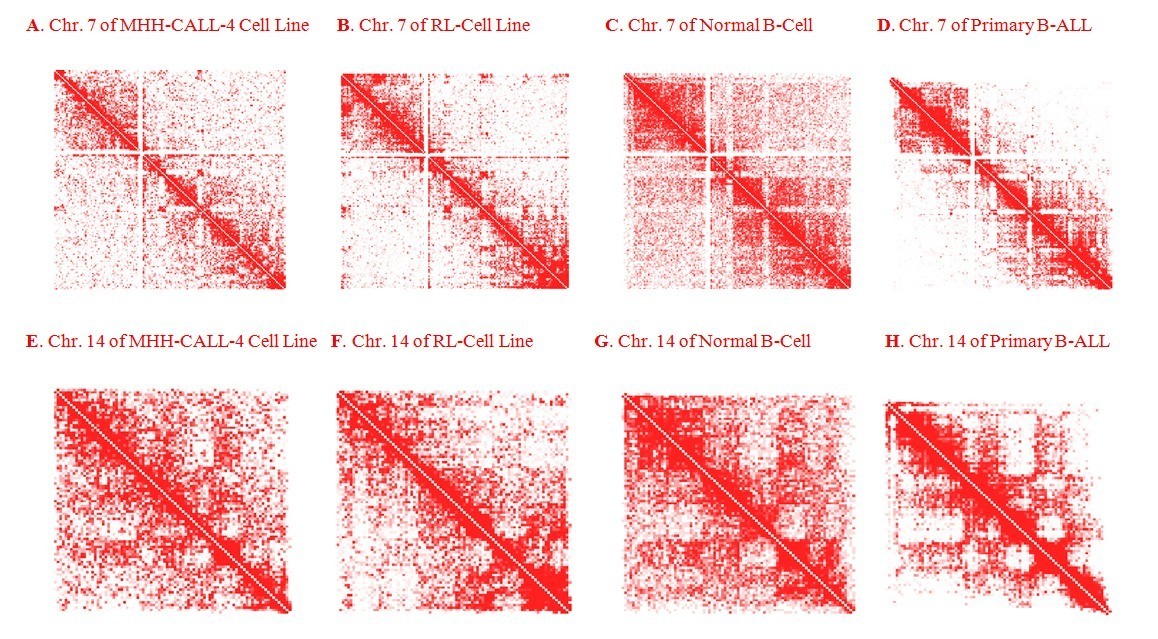

Supplement: Figure S14 — Contact significance analysis of selected chromosomes. In order to check if the number of contacts between two specific chromosome regions is significantly large, we calculated the significance score (i.e. the probability of receiving this number of contacts or more) in each cell of an intra-chromosome contact matrix at 1Mb resolution, assuming the background distribution of contact numbers follows the Poisson distribution. The parameter (lamda: mean contact number) of the background distribution was set to the average of number of contacts in the matrix excluding contacts within the same region (i.e. diagonal line in a matrix). Sub-figures A, B, C and D illustrate the contact significant scores of the intra-chromosomal contract matrices of chromosome 7 of the MHH-CALL-4 cell line, RL cell line, normal B-cell and the Primary B-ALL cell, respectively. Sub-figures E, F, G and H depict the significance scores of intra-chromosomal contact matrices of chromosome 14 of the MHH-CALL-4 cell line, RL-cell line, normal B-cell line and the primary B-ALL cell, respectively. Darker red indicates higher significant score. (JPG) [file pone.0058793.s014.jpg]

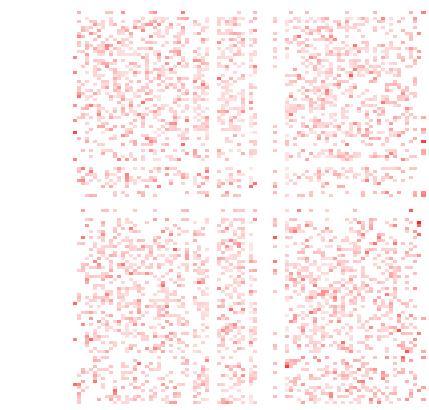

Supplement: Figure S15 — The inter-chromosome contact matrix between chromosome 11 and 14 of Normal B-Cell Line. (JPG) [file pone.0058793.s015.jpg]

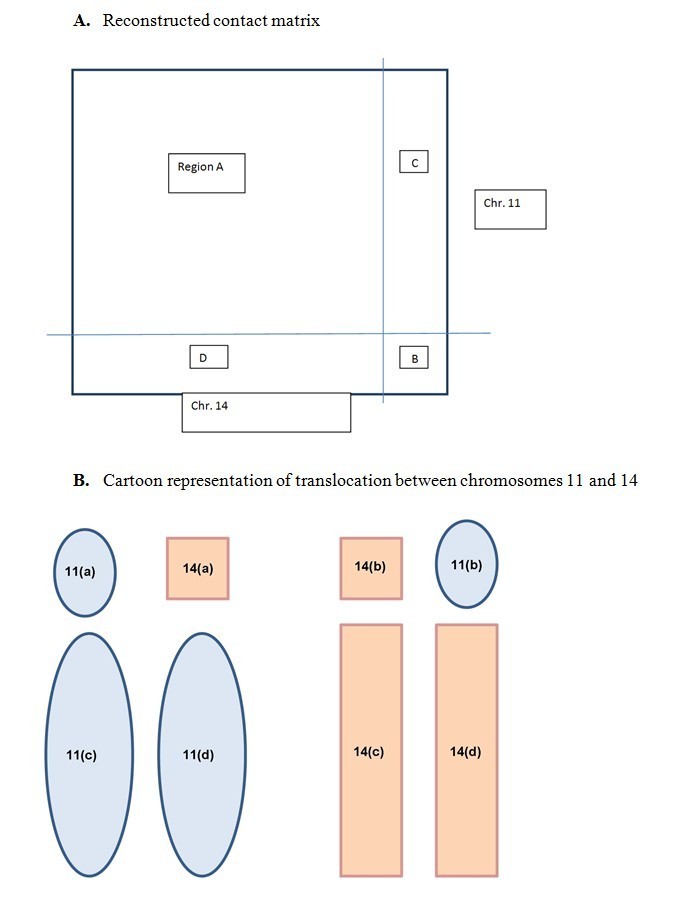

Supplement: Figure S16 — The method of calculating the corrected inter-chromosomal contact matrix of translocated chromosomes. (A) Division of the corrected inter-chromosomal contact matrix between chromosomes 11 and 14 into three regions to be reconstructed separately. Region A contains the contacts between non-translocated segments in chromosome 11 (i.e. 11(c) and 11(d) in (B)) and non-translocated segments in chromosome 14 (i.e. 14(c) and 14(d) in (B)). Region B contains the contacts between translocated segments in chromosome 11 (i.e. 11(b) in (B)) and translocated segments in chromosome 14 (i.e. 14(a) in (B)). Region C contains the contacts between non-translocated segments in chromosome 11 (i.e. 11(c) and 11(d) in (B)) and translocated segments in chromosome 14 (i.e. 14(a) in (B)). Region D contains the contacts between non-translocated segments in chromosome 14 and translocation segment in chromosome 11. For the contacts in regions A and B, we divided the original contact numbers by 2 in order to estimate the inter-chromosome contacts. For region C, we normalized the value of each cell Cij = max (0, Cij – average num of row i in region A). For region D, we normalized the value of each cell Dij = max(0, Dij – average num of column j in region A). (JPG) [file pone.0058793.s016.jpg]

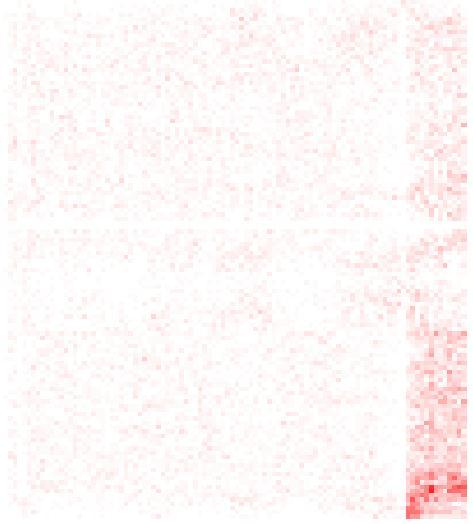

Supplement: Figure S17 — The corrected inter-chromosomal contact map between translocated chromosomes 11 and 14 for the primary ALL B-cell. The method of calculating it can be found in Figure S13. (JPG) [file pone.0058793.s017.jpg]

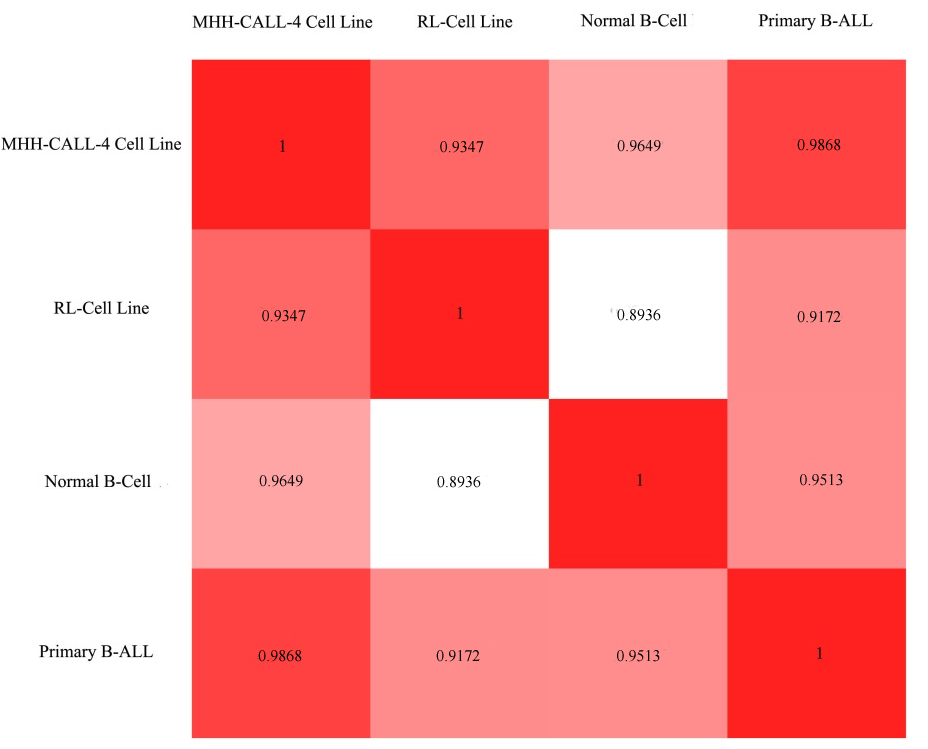

Supplement: Figure S18 — The Pearson’s correlation matrix for inter-chromosomal contact numbers between the normal B cell, primary ALL B-cell, MHH-CALL-4 cell line, and RL cell line. For each cell, the number of inter-chromosomal contacts between chromosomes were calculated and put into a vector. Thus, each cell has one vector to represent all its inter-chromosomal contact numbers. The matrix below shows the Pearson’s correlation between each pairs of vectors of two cell samples. (JPG) [file pone.0058793.s018.jpg]

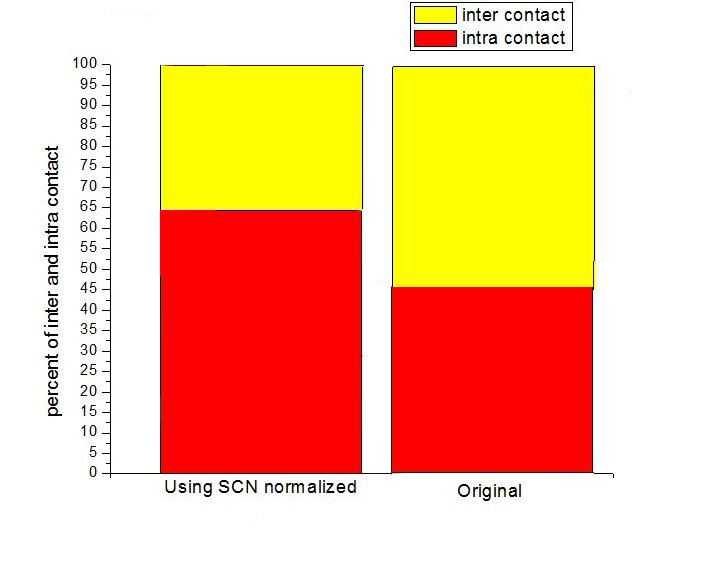

Supplement: Figure S19 — The distribution of inter and intra chromosome contact number for Normal-B Cell before using SCN procedure and after using SCN procedure. The number of inter contact after using SCN is calculated by summing up all inter chromosome contact matrix normalized by using SCN procedure, and then divided by the number of inter chromosome contact matrix. The number of intra contact after using SCN is calculated by summing up all intra chromosome contact matrix normalized by using SCN procedure, and then divided by the number of intra chromosome contact matrix. (JPG) [file pone.0058793.s019.jpg]

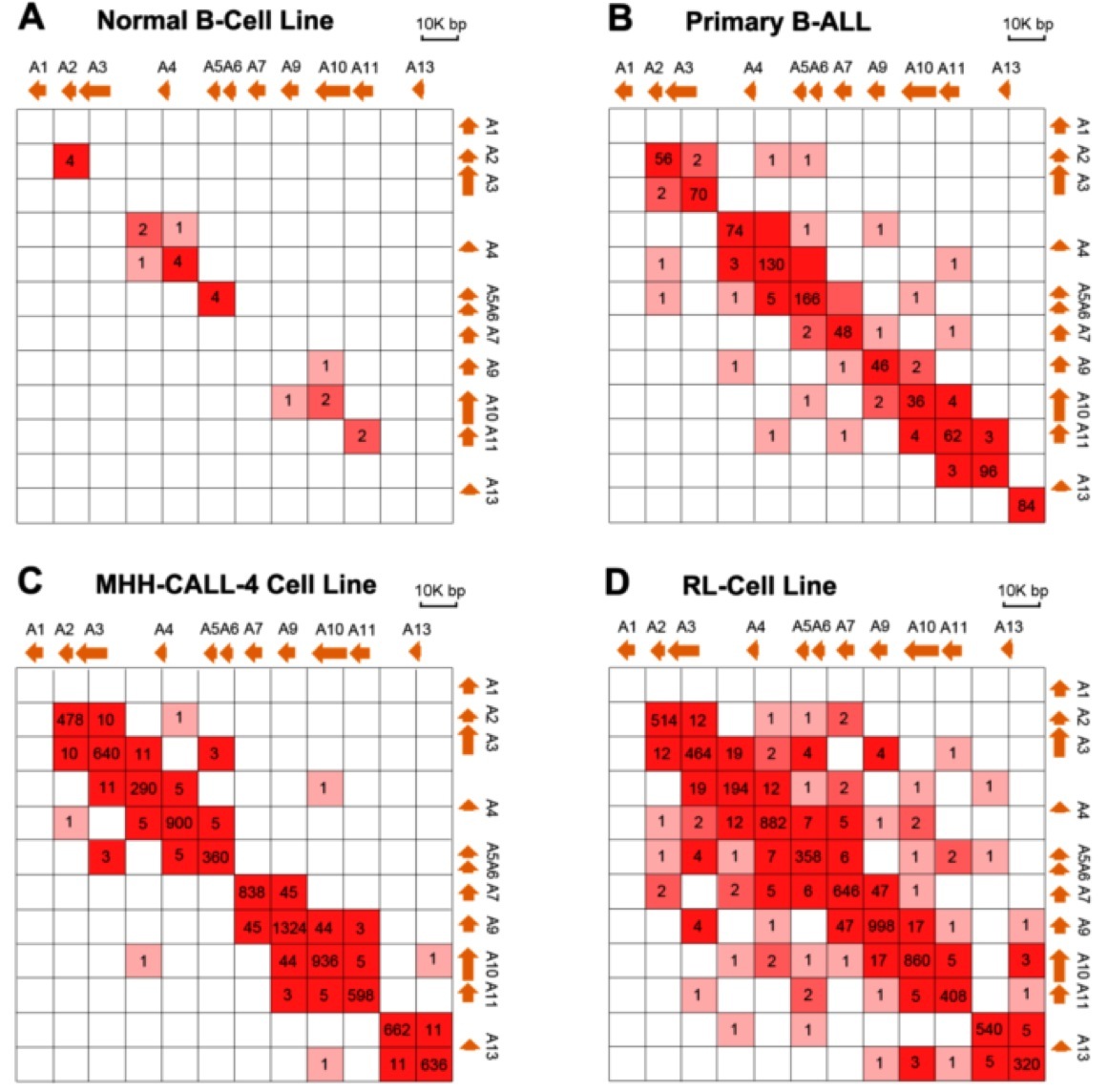

Supplement: Figure S20 — The number of contacts between 13 genes in the cluster in each cell line. (JPG) [file pone.0058793.s020.jpg]

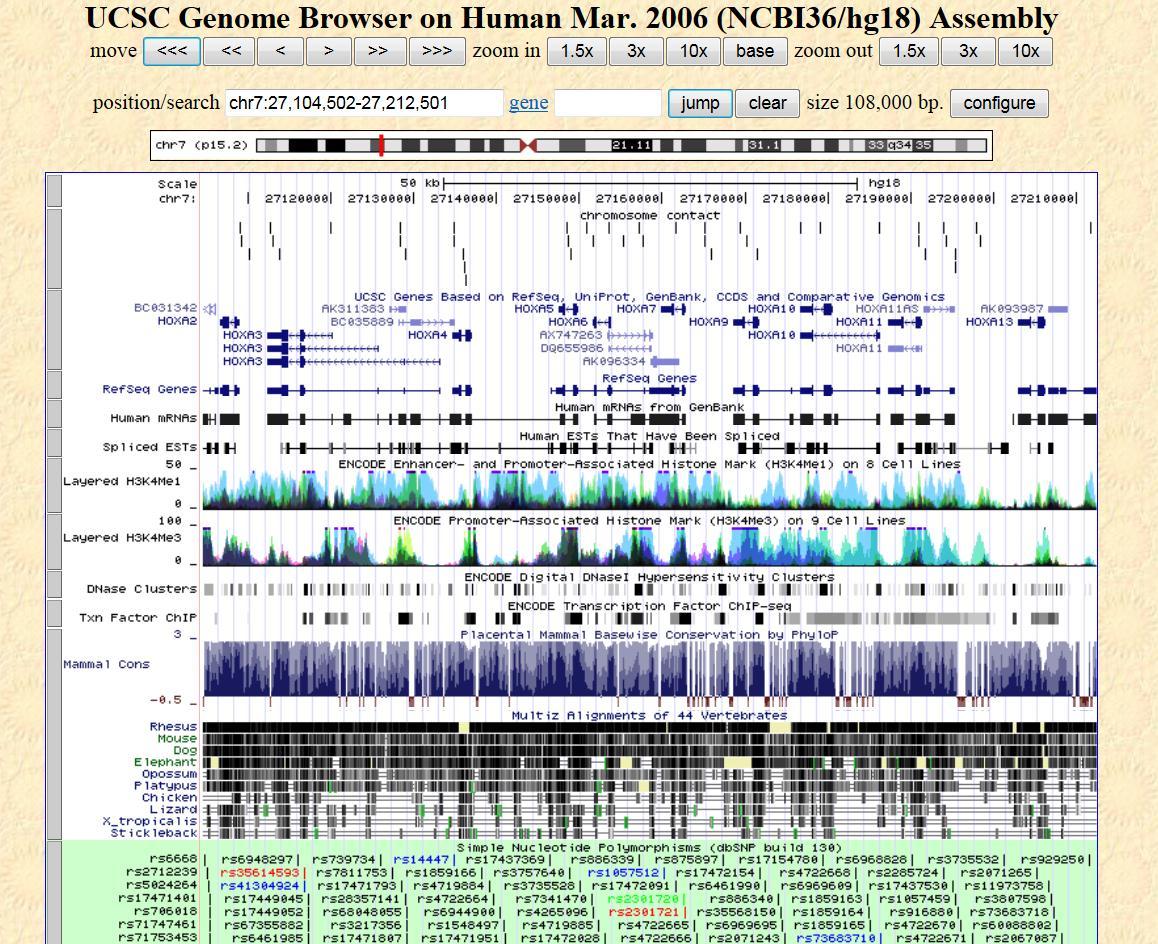

Supplement: Figure S21 — The visualization of reads mapped to the HoxA gene region (27,104,502 – 27,212,501) on chromosome 7 of the human genome by the UCSC genome browser. The vertical line segments under the label “chromosome contact” denote the locations where the reads were mapped to. The reads data of the MHH-CALL-4 cell line was used. (JPG) [file pone.0058793.s021.jpg]

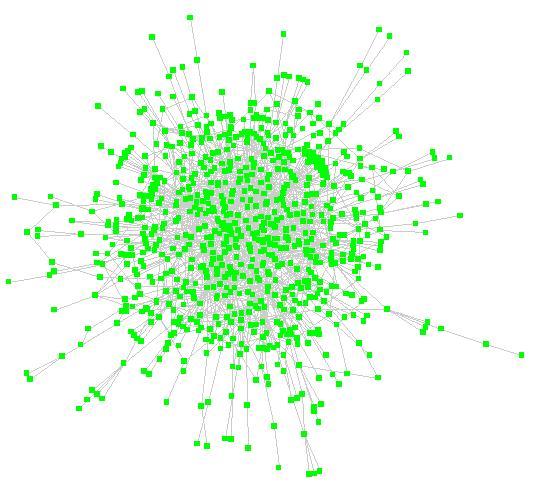

Supplement: Figure S22 — The networks of inter-chromosomal gene-gene interactions between chromosome 11 and 14 for RL-Cell Line. (JPG) [file pone.0058793.s022.jpg]

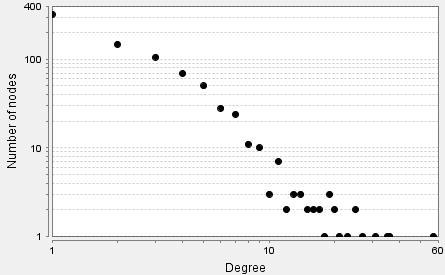

Supplement: Figure S23 — Node-degree distribution of Figure 22. (JPG) [file pone.0058793.s023.jpg]

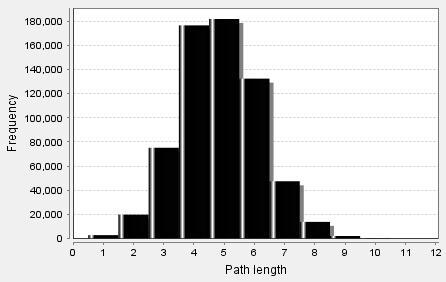

Supplement: Figure S24 — Shortest path frequency of Figure 22. (JPG) [file pone.0058793.s024.jpg]

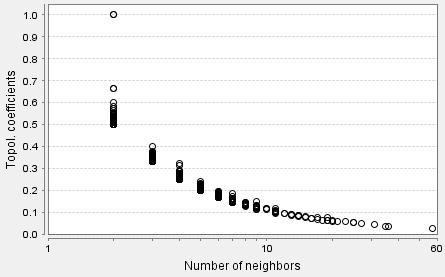

Supplement: Figure S25 — The distribution of topological coefficients of the networks shown in Figure 22. (JPG) [file pone.0058793.s025.jpg]

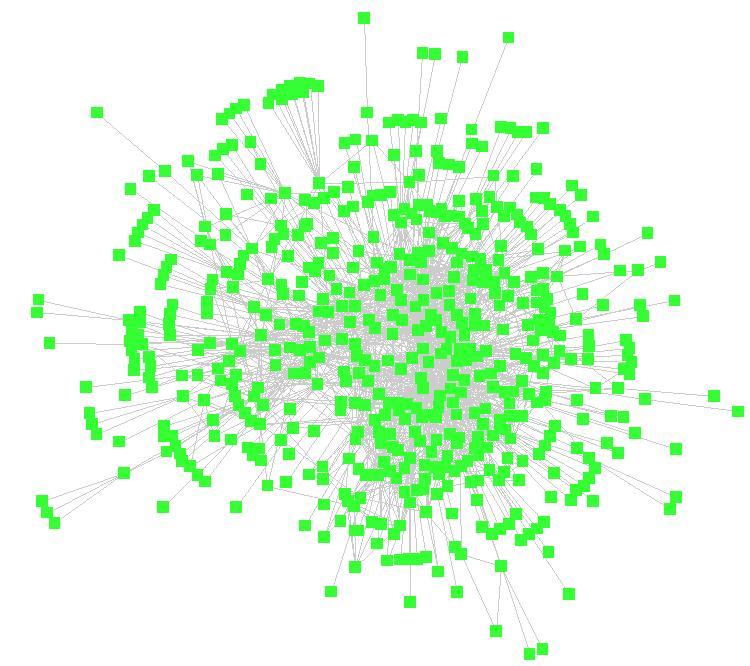

Supplement: Figure S26 — The interaction network between transcription factor binding sites (TBSs) in the entire genome of the MHH-CALL-4 cell line. This is generated based on raw contacts. (JPG) [file pone.0058793.s026.jpg]

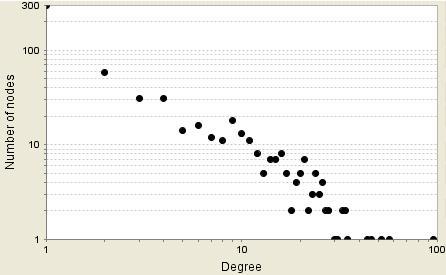

Supplement: Figure S27 — The distribution of node degree of the TBS-TBS interaction network shown in Figure S26. (JPG) [file pone.0058793.s027.jpg]

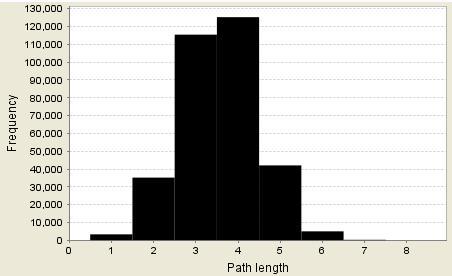

Supplement: Figure S28 — The histogram of lengths of the shortest paths between any two nodes in the TBS-TBS interaction network shown in Figure S26. (JPG) [file pone.0058793.s028.jpg]

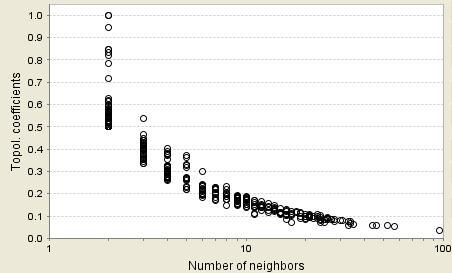

Supplement: Figure S29 — The distribution of topological coefficients the TBS-TBS interaction network shown in Figure S26. (JPG) [file pone.0058793.s029.jpg]

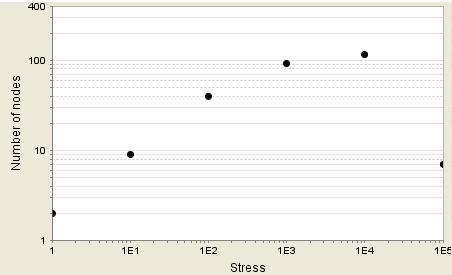

Supplement: Figure S30 — The distribution of node stresses of the TBS-TBS interaction network shown in Figure S25. (JPG) [file pone.0058793.s030.jpg]

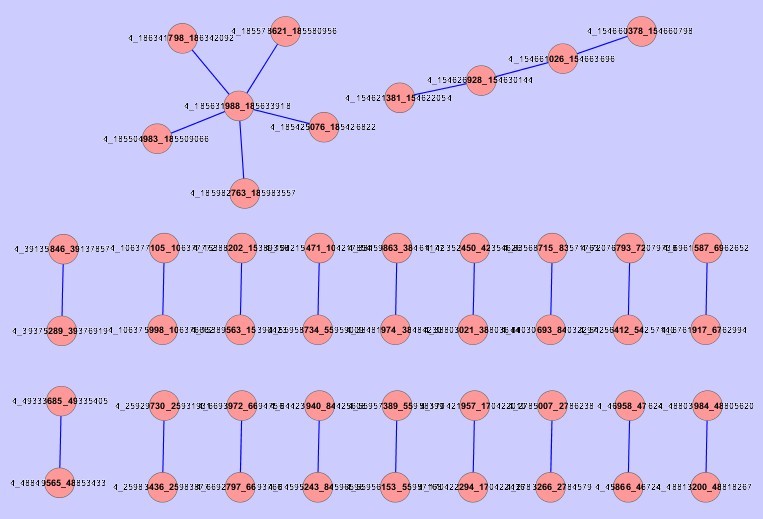

Supplement: Figure S31 — The interaction network between transcription factor binding sites (TBSs) of chromosome 4 and chromosome 4 of the MHH-CALL-4 cell line. This network is normalized with the contact threshold 2. (JPG) [file pone.0058793.s031.jpg]

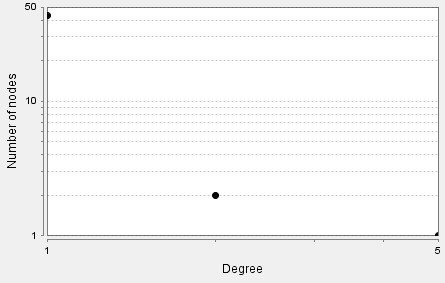

Supplement: Figure S32 — The distribution of node degree of the TBS-TBS interaction network shown in Figure S31. (JPG) [file pone.0058793.s032.jpg]

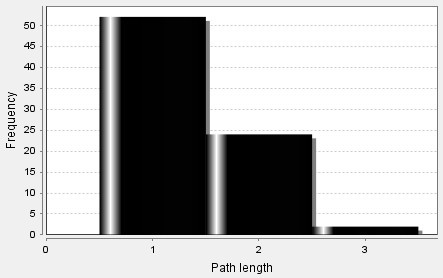

Supplement: Figure S33 — The histogram of lengths of the shortest paths between any two nodes in the TBS-TBS interaction network shown in Figure S31. (JPG) [file pone.0058793.s033.jpg]

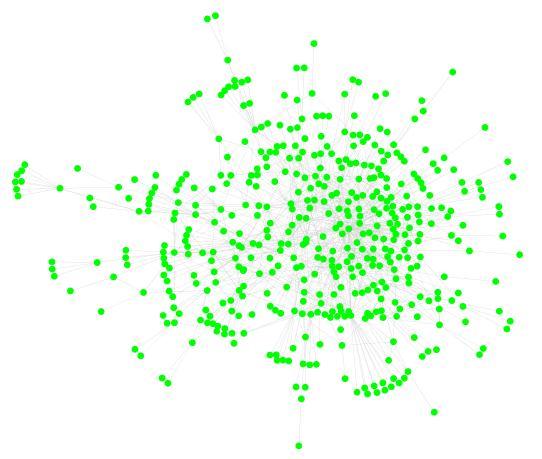

Supplement: Figure S34 — The spatial interaction networks between genes and transcription factor binding sites (TFB) in chromosome 14 for the CALL-4 cell line. A node in the network denotes a gene or a TFB. Two nodes are connected by an edge if they are spatially contacted. (JPG) [file pone.0058793.s034.jpg]

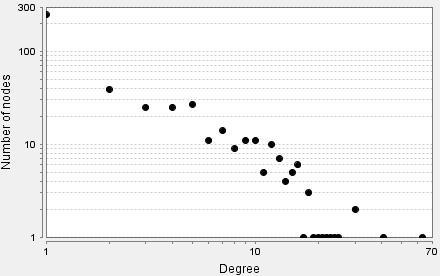

Supplement: Figure S35 — The node degree distribution of the network shown in Figure S34. It is shown that the frequency (number of nodes) is largely linear to the degree of the nodes on the log-log scale. This suggests that the network is likely a scale-free network. (JPG) [file pone.0058793.s035.jpg]

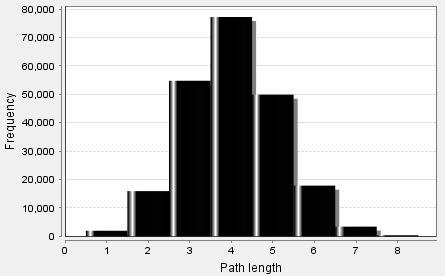

Supplement: Figure S36 — The histogram of lengths of the shortest path between any two nodes in the network shown in Figure S34. (JPG) [file pone.0058793.s036.jpg]

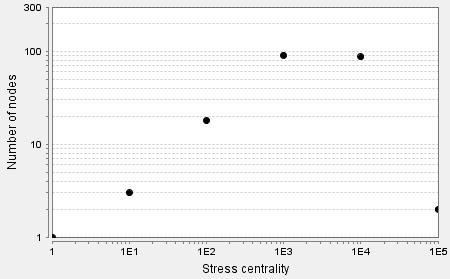

Supplement: Figure S37 — The distribution of stress values of the network shown in Figure S34. (JPG) [file pone.0058793.s037.jpg]

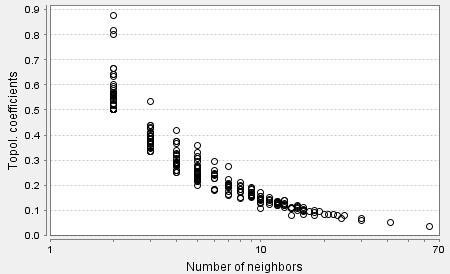

Supplement: Figure S38 — The distribution of topological coefficients of the network shown in Figure S34. (JPG) [file pone.0058793.s038.jpg]

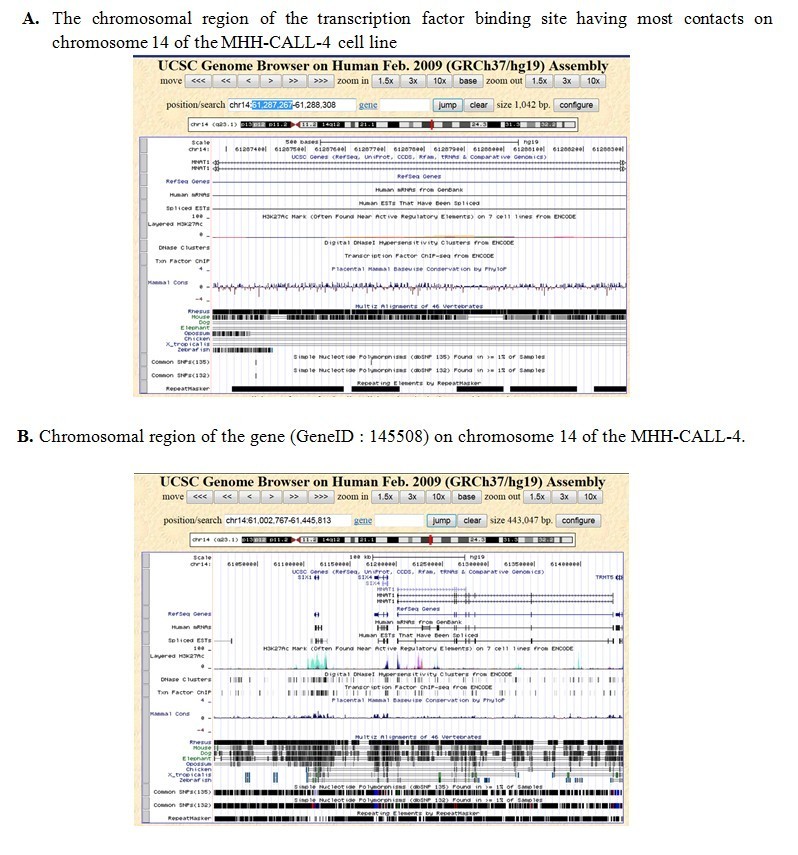

Supplement: Figure S39 — (A) The chromosomal region of the transcription factor binding site on chromosome 14 of the MHH-CALL-4 cell line that has the highest contacts with other genes is visualized by the UCSC genome browser. This transcription factor binding site contacted 1460 times with GeneID:145508 (starting from the position 61002767 and ending at 61445813), 1 time with GeneID:7253, 2 times with GeneID:6710, 2 times with GeneID:56659, and 1 time with GeneID:9369. (B) The chromosomal region of the gene (GeneID:145508) that encodes a centrosomal protein (128 kDa). More information about this gene is available at http://www.ncbi.nlm.nih.gov/sites/entrez?db=gene&term=145508. (JPG) [file pone.0058793.s039.jpg]

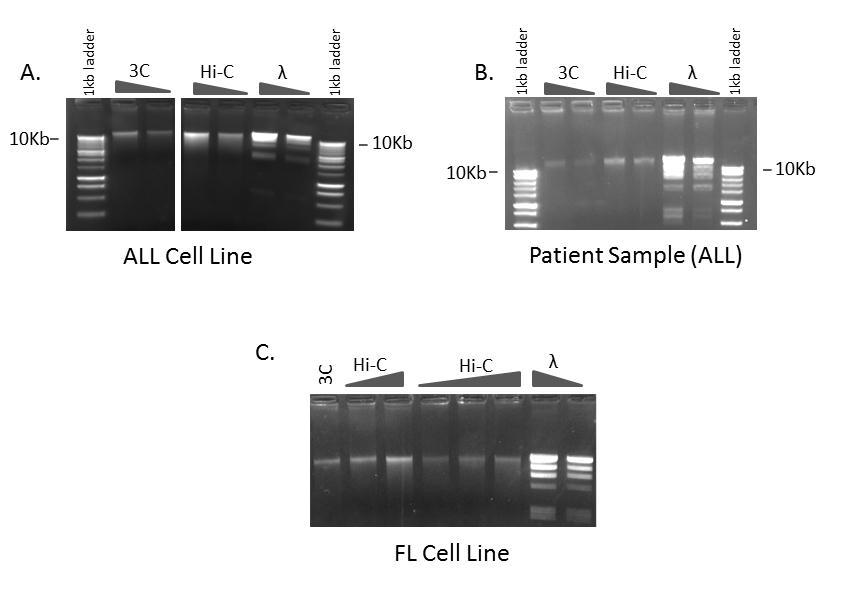

Supplement: Figure S40 — Ligation efficiency. Both the 3C and Hi-C libraries should run as a fairly tight band larger than 10 Kb. Ligation efficiency is slightly lower in Hi-C than in 3C and is indicated by the smear in the Hi-C lanes (see van Berkum et al. in JoVE for a complete description). The right triangles above each panel represent increasing or decreasing amounts of template. The Lambda HindIII ladder (λ) and a 1 Kb DNA ladder were also included on the visualization gels. The uppermost fragment of the lambda HindIII ladder is 23.13 Kb and the uppermost fragment of the 1 Kb ladder is 10 Kb. A quantative agarose gel was run (0.8%) on an acute lymphoblastic leukemia cell line (A), an acute lymphoblastic leukemia patient sample (B) and a follicular lymphoma cell line (C). Panel C includes 5 Hi-C template amounts and one 3C template amount whereas panels A and B both include 2 Hi-C and 2 3C template amounts. (JPG) [file pone.0058793.s040.jpg]

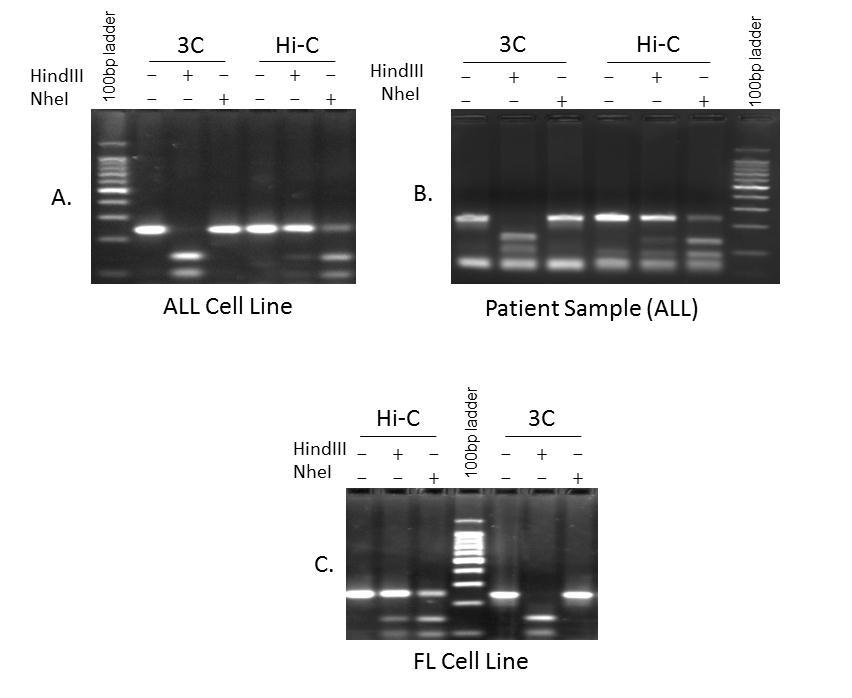

Supplement: Figure S41 — PCR digest control. A quantative agarose gel was run (0.8%) on an acute lymphoblastic leukemia cell line (A), an acute lymphoblastic leukemia patient sample (B) and a follicular lymphoma cell line (C). During Hi-C the HindIII site is lost and an NheI site is created (see van Berkum et al. in JoVE for a complete description of the protocol) and the products can be distinguished from a 3C experiment by digesting the ligation site. The digested samples were quantified using ImageJ software. A total of three PCR reactions were done for both 3C and Hi-C samples. The reactions were pooled and purified using the Zymo clean and concentrator kit per the manufacturer’s protocol eluting 2x with 10 µL with water. The PCR products were digested with HindIII (no blunting of the DNA ends) or NheI (shows blunting and biotin incorporation). 56% (FL Cell Line), 64% (ALL Cell Line) and 68% (Patient Sample) of Hi-C amplicons were digested by NheI confirming the efficient marking of ligation junctions. (JPG) [file pone.0058793.s041.jpg]

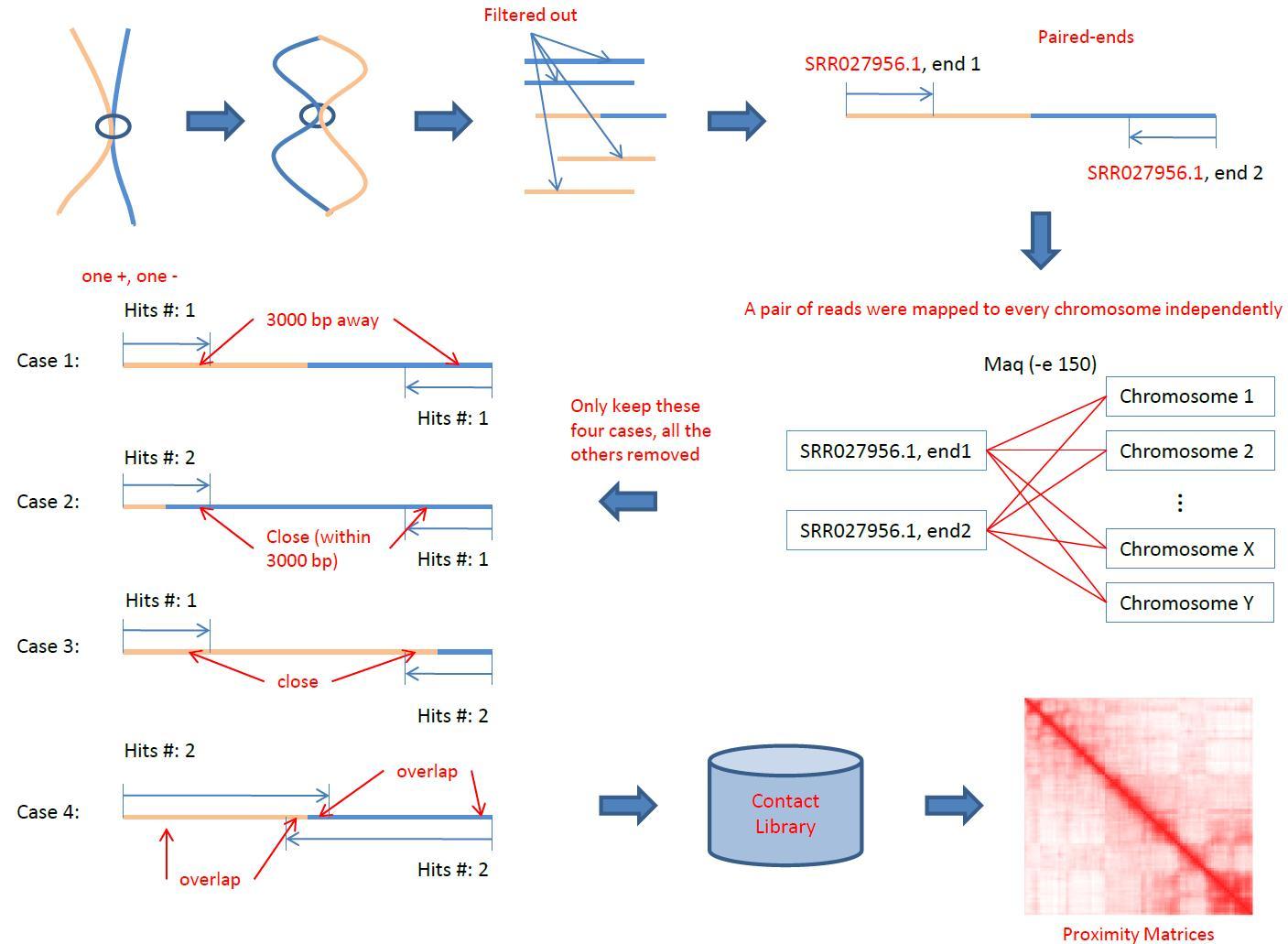

Supplement: Figure S42 — An overview of the bioinformatics pipeline of analyzing Hi-C experimental reads data. The Hi-C wet lab experiment is similar to the method described in [13], in which chromosome DNA is cross-linked, ligated and then sheared. Each of the reads-pairs was mapped to the human genome by the tool maq (http://maq.sourceforge.net/) with the mistake threshold (–e) set to 150. Our computer programs analyzed the mapping output and handled the four different cases in which the reads may cover different portions of the two chromosomes. These four situations are illustrated in the following figure. Case one is that each of the two ends can only be mapped to one location and the two mapped locations of the two ends are 2000b away. Case two is one end can be mapped to one location (e.g. location A), but the other end to two locations (e.g. B and C). In this case, we checked whether one of the two locations (B or C) is within 2000b of A. If not, the case is considered invalid and is discarded. 2000bp was used as the threshold because the average length of the DNA insert is 2000bp long. Case three is the same as Case two except that the first end was mapped to two locations and the second to one location. Case four is both two ends can be mapped to two locations (e.g. one end to A and B, and the other to C and D as shown in the figure. A, B, C, and D are the starting positions of the mapping locations). In this case, we checked whether the distance between A and C is less than the read length and whether the distance between B and D is less than the read length. If yes, they were kept. In our first mapping strategy, only these four cases above were considered and processed to generate contacts and all the other cases were discarded. For example, if one end of a pair of ends can be mapped to > = 3 locations, they were discarded. This process was able to reduce noise (e.g. wrongly-aligned reads) and ensure the quality of contact parsing. We also developed the second simplified strategy [file pone.0058793.s042.jpg]

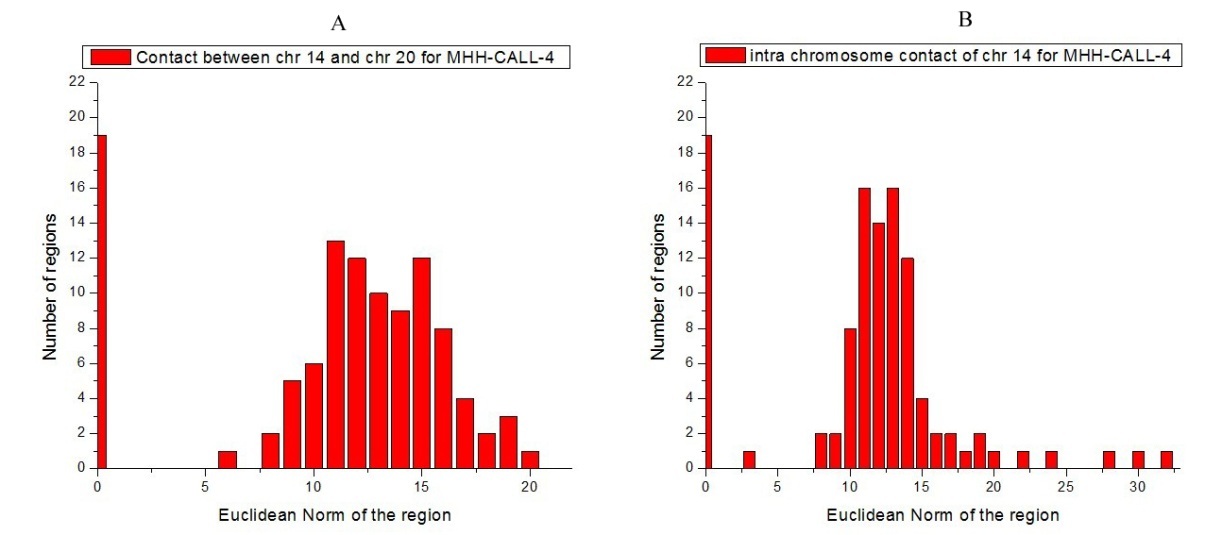

Supplement: Figure S43 — The distribution of Euclidean Norm of each region of both inter and intra chromosome contact for MHH-CALL-4. Figure S37(A) shows the distribution of Euclidean Norm of each region of chromosome 14 which has inter chromosome contact with chromosome 20 of MHH-CALL-4, we can see a Gaussian distribution for the Euclidean Norm, and we set 10 as a threshold, all regions which have Euclidean Norm less than 10 will be removed. The resolution for each region is 1M. Figure S37 (B) shows the distribution of Euclidean Norm of the intra chromosome contact of chromosome 14 of MHH-CALL-4. The resolution for each region is 10M. We set 10 as the threshold to normalize the contact map. The threshold 100 is set to normalize the intra contact map when the resolution for each region is 1M. (JPG) [file pone.0058793.s043.jpg]
