# Supplementary material for: The Properties of Genome Conformation and Spatial Gene Interaction and Regulation Networks of Normal and Malignant Human Cell Types
Source: PLoS One. 2013 Mar 11;8(3):e58793. doi: 10.1371/journal.pone.0058793 (PMC3594155; doi:10.1371/journal.pone.0058793)
Supplement: Table S1 — Reads coverage of the gene regions and non-gene regions. The reads coverage of gene region was calculated as the reads length multiply the number of contact in gene region/total length of gene region. The coverage of non-gene region was calculated as the read length * number of contact not in gene region/total length of non-gene region. Here the gene length was calculated according to the gene start and end information. (DOCX) [file pone.0058793.s044.docx]

|  | Read coverage of gene region | Read coverage of non-gene region | Read length |
| --- | --- | --- | --- |
| Call4 cell line | 2.81129121903121 | 2.35780895 | 100 |
| RL cell line | 1.47413416423764 | 1.14648699 | 100 |
| Normal B-cell | 0.186290512630489 | 0.1725446 | 76 |
| ALL B-cell | 1.788587532589 | 1.49037874 | 120 |
