# Supplementary material for: The Properties of Genome Conformation and Spatial Gene Interaction and Regulation Networks of Normal and Malignant Human Cell Types
Source: PLoS One. 2013 Mar 11;8(3):e58793. doi: 10.1371/journal.pone.0058793 (PMC3594155; doi:10.1371/journal.pone.0058793)
Supplement: Table S2 — Total number of reads for all samples mentioned in this work. The data of the normal B cell was downloaded from the publication [13]. The others were generated by us. One pair-end read pair contains two ends of reads. This table shows the total number of ends. For some cell/cell lines, we sequenced them more than one times and selected the one with the best quality to use in this work. (DOCX) [file pone.0058793.s045.docx]

| **Samples** | **Total number of reads** | **Utilized for analysis** |
| --- | --- | --- |
| Normal B cell | 12,887,282 | YES |
| RL1 | 60,272,006 | NO |
| RL2 | 61,043,078 | NO |
| RL3 | 65,579,872 | NO |
| RL4 | 125,256,746 | YES |
| Call4_1 | 62,741,712 | NO |
| Call4_2 | 62,607,906 | NO |
| Call4_3 | 133,542,778 | YES |
| ALL B-Cell | 77,888,742 | YES |
