# Supplementary material for: The Properties of Genome Conformation and Spatial Gene Interaction and Regulation Networks of Normal and Malignant Human Cell Types
Source: PLoS One. 2013 Mar 11;8(3):e58793. doi: 10.1371/journal.pone.0058793 (PMC3594155; doi:10.1371/journal.pone.0058793)
Supplement: Text S1 — The description of materials and methods of the Hi-C experiment. (DOCX) [file pone.0058793.s046.docx]

**HI-C Procedure (**adapted from Erez Lieberman-Aiden, et al. *Comprehensive Mapping of Long-Range Interactions Reveals Folding Principles of the Human Genome*, *Science* 326, 289 (2009))

*Crosslinking of Cells.* In a 15 mL conical tube 25 million cells were centrifuge for 5 minutes at 300xg. The cell pellets were resuspended in 45 mL of Media. To crosslink the DNA 1.25mL of 37% formaldehyde was added for a final concentration of 1%. Then the solution was incubated for 10 minutes at room temperature. To stop the reaction 2.5 mL of 2.5 M glycine was added and incubated for 5 minutes at room temperature. Subsequently tubes were placed on ice for 15 minutes. The cells were collected by centrifuging at 1,500rpm for 10 minutes. After the supernatants were discarded the pellets were stored at -80^o^C until further processed.

*Cell lysis and chromatin digestion.* The cell pellet was resuspended in 500 µL of lysis buffer (10 mM Tris-HCl pH8.0, 10 mM NaCl, 0.2% Ige pal CA630), and 50 µL of protease inhibitor was added. After which it was incubated on ice for 25 minutes. The cell suspension was transferred to a 1.5 mL microcentrifuge tube. To collect the crosslinked DNA the tube was centrifuged at 5,000 rpm for 5 minutes. The pellet was washed twice with ice-cold 1xNEBuffer 2, and resuspended in 250 µL of 1xNEBuffer 2. Then 50 µL was aliquoted into 5 separate microcentrifuge tubes. For each tube, 312 µL of 1xNEBuffer2 was added. To remove the non-crosslinked proteins 38 µL of 1% SDS was added followed by incubation for 10 minutes at 65^o^C. To quench the SDS, 44 µL of 10% Triton X-100 was added. Followed by 20 µL of HindIII(400units) to digest the DNA. The tubes were incubated at 37^o^C overnight with rotation.

*Marking of DNA end and blunt-end ligation.* All of the tubes were placed on ice. One of the five is set aside to be the 3C control. To fill in and mark the DNA ends the following was added the other four tubes: 1.5 µL 10 mM dATP, 1.5µL 10 mM dGTP, 1.5 µL 10 mM dTTP, 37.5 µL 0.4 mM biotin-14-dCTP, and 10 µL 5 units/µL Klenow. The mix was incubated at 37^o^C for 45 minutes and cooled on ice. To inactivate the enzyme 86 µL of 10% SDS was added and incubated at 65^o^C for 30 minutes then cooled on ice. For dilute blunt-end ligation five 15 mL conical tubes were prepared by adding 7.61 mL of ligation mix (745 µL of 10% Triton X-100, 745 µL 10x ligation buffer (500 mM Tris-HCl pH7.5, 100 mM MgCl2, 100 mM DTT), 80µL 10 mg/mL BSA, 80 µL 100 mM ATP and 5.96 of water). Each of the five microcentrifuge tubes digested with HindIII are added to one 15 mL conical tube. For the 3C control 10 µL 1 unit/µL of T4 DNA ligase was added, and for the four tubes of HI-C 50 µL of 1 unit/µL T4 DNA ligase was added. The tubes were then incubated for six hours in a circulating water bath at 16^o^C.

*DNA Purification.* To degrade the protein 25 µL of 20mg/mL proteinase K was added to each tube and incubated at 65^o^C overnight. An additional 25 µL of 20 mg/mL was added and incubated at 65^o^C for two hours. Then the tubes were allowed to cool to room temperature and transferred to 50 mL conical tubes. The DNA was isolated by the addition of 10mL of phenol pH8.0 and vortexed for two minutes, and then the sample was centrifuged at 3,500 rpm for ten minutes. The aqueous phase was transferred to a new 50 mL conical tube. A second isolation was done by adding 10mL of phenol:chloroform (1:1) and vortexed for two minutes. Subsequently the sample was centrifuged at 3,500 rpm for ten minutes. The aqueous phase was transferred to a new 50mL conical tube, and the total volume was brought up to 10 mL with 1x TE. To precipitate the DNA, 1 mL of 3 M Na-acetate and 25 mL of ice-cold 100% ethanol was added and mixed by vortexing. Followed by an incubation at -80^o^C for at least one hour. To pellet the DNA, the tubes were centrifuged at 3,000 xg for 40 minutes at 4^o^C. The pellets were then dissolved in 450 µL of 1xTE and transferred to a 1.5 mL microcentrifuge tube. The DNA was extracted twice by adding 500 µL of phenol:chloroform (1:1), vortexed for 30 seconds and centrifuged at ≥13,000 rpm for five minutes at room temperature. The aqueous phase of was transferred to a new tube. After the second extraction, to precipitate the DNA, 40 µL of 3 M Na-acetate and 1 mL of 100% ice-cold ethanol was added then vortexed and incubated at -80^o^C for at least 30 minutes. To pellet the DNA the tubes were centrifuged at ≥13,000 rpm for 20 minutes at 4^o^C. The pellet was washed once with 70% ethanol and centrifuged at ≥13,000 rpm for five minutes at 4^o^C. After the supernatant is discarded the pellet was air dried for 10 minutes. Followed by a resuspention in 50 µL of 1x TE. To remove the RNA, 2 µL of 1 mg/mL RNAse was added and incubated at 37^o^C for 15 minutes. All of the HI-C tubes were combined and the 3C was kept separate.

*Quality control for the HI-C libraries.* A 0.8% agarose quantative gel was ran on both the 3C and HI-C samples with the lambda HindIII ladder, and quantified using ImageJ software. To verify ligation efficiency a PCR was ran as follows: 2.5 µL of 10x amplitaq gold buffer, 2.5 µL 2.5 mM MgCl, 1µL 400 µM each dNTP, 0.75 µL 0.3µM forward primer, 0.75µL 0.3 µM reverse primer, 0.625 units amplitaq gold, 25 ng template, and water up to 25 µL. The PCR primer sequences used were 5’-gttcatcttgctgccagaaatgccgagcctg-3’; 5’-atcccagctgtctgtagctttagaaagtggg-3’. These primers are both on the positive strand thus if there is amplification in the PCR then there has been interaction other than religating of the pieces back together. The PCR conditions used are as follows: 95^o^C for 12min; 3 cycles of 95^o^C for 45 sec, 68^o^C for 45 sec (a step down one degree each cycle), 72^o^C for 1 min; 34 cycles of 95^o^C for 45sec, 65^o^C for 45 sec, 72^o^C for 1 min; 72^o^C for 10 min. Three PCR reactions were done For both 3C and HI-C samples. The reactions were pooled and purified using the Zymo clean and concentrator kit per the manufacturer’s protocol eluting 2x with 10 µL with water. Then the PCR products are digested with HindIII (no blunting of the DNA ends) or NheI (shows blunting and biotin incorporation). The expected incorporation yield is 50% to 70%.

*Removal of biotin from unligated ends.* Using the exonuclease activity of T4 DNA polymerase the biotin-14-dCTP was removed from the unligated DNA ends. This was done by adding 5ug of purified DNA, 1 µL 10 mg/mL BSA, 10 µL 10x NEBuffer 2, 1 µL 10 mM dATP, 1 µL 10 mM dGTP, 5 units T4 DNA polymerase, and water up to 100 µL in PCR tubes. Two reactions were done for each sample of HI-C. The tubes were incubated at 12^o^C for two hours in a thermal cycler and stopping the reaction with 2 µL of 0.5M EDTA pH8.0. Phenol/Chloroform extraction and ethanol precipitation was done on the samples and the pellets were resuspended in 25 µL of water, combining pellets if multiple were done, and the total volume was brought up to 100µL in water.

*Shearing and size selection.* The DNA was sheared to a size of ~300-500 bp using a biorupter by sonicating for eight minutes, 30 sec on and 30 sec off, and then four more minutes of 30 sec on and 30 sec off. End repair was carried out by adding the following, 14 µL 10x NEB ligation buffer, 14 µL 2.5mM dNTP mix, 5 µL T4 DNA polymerase, 5 µL T4 polynucleotide kinase, 1 µL Klenow DNA polymerase, and 1 µL water to the sheared DNA. The tubes were incubated at 20^o^C for 30 minutes and purified using the Qiagen MinElute column per the manufacturer’s protocol eluting twice with 15 µL of 1x TE. To add an ‘A’ to the 3’ end the following was added to the 30 µL of purified DNA; 5 µL 10x NEBuffer2, 10 µL 1 mM dATP, 3 µL Klenow (exo-), and 2 µL of water. The tubes were incubated at 37^o^C for 30 minutes, and then at 65^o^C for 20 minutes to inactivate the Klenow. Samples were cooled on ice and the volume reduced to ~20 µL using a speedvac. Gel purification was conducted by running and excising the 300-500 bp and purified using the Qiagen gel extraction kit per the manufacturer’s protocol eluting twice with 50 µL of 1xTLE (Tris Low EDTA; 10 mM Tris pH8.0, 0.1 mM EDTA). The Final volume was brought up to 300 µL with 1x TLE.

*Biotin pulldown and Paired End sequencing.* All of the following steps were done in 1.5 mL microcentrifuge DNA LoBind tubes. Streptavidin beads were mixed and 150 µL/sample was transferred to the LoBind tube. Wash steps are done by adding the buffer to the beads, transferring to a new tube, rotating the sample for three minutes at room temperature, reclaiming the beads using a magnet for one minute, and removing the supernatant. Beads were washed twice with 400 µL of Tween Wash Buffer (TWB; 5 mM Tris-HCl pH8.0, 0.5 mM EDTA, 1 M NaCl, 0.05% Tween-20). Then they were resuspended in 300 µL of 2x Binding Buffer (BB; 10 mM Tris-HCl pH8.0, 1 mM EDTA, 2 M NaCl). To the beads, the 300 µL of DNA after it was size selected was added. Afterward the beads plus DNA was incubated at room temperature for 15 minutes with rotation. Beads were collected with a magnetic rack for one minute, and washed with 400 µL of 1x BB, 100 µL of 1x ligation buffer, and finally 50 µL of 1x ligation. To ligate the paired end adapters add 4 µL of 6 picomoles/ug of paired end adapters and 3 µL (1200 units) of T4 DNA Ligase. The tubes were incubated for two hours at room temperature. To remove the non-ligated adapters the beads were washed with 400 µL of 1x TWB, 200 µL of 1x BB, 1x NEBuffer2, and then 50 µL of 1x NEBuffe2. The pellet was resuspended in 50 µL of NEBuffer2. Test PCR reactions were done to find the optimal PCR cycles to generate enough library for sequencing. The PCR reactions were set up using 0.6 µL of HI-C Streptavidin bead bound DNA, 1 µL of PE1.0 PCR primer (1.5 pmol), 1 µL PE2.0 PCR primer (1.5 pmol), 8 µL 2x Phusion High Fidelity master mix, and 4.4 µL of water. Cycling conditions were 98^o^C for 30 seconds; 12, 17, or 21 cycles of 98^o^C for 10 seconds, 65^o^C for 30 seconds, 72^o^C for 30 seconds; and 72^o^C for 7 minutes. The number of cycles was chosen that had a smear between 400bp and 600bp and had enough library for sequencing. That number of cycles was used in the large scale PCR which was done in 4x 50 µL reactions with 40 µL of HI-C Streptavidin bead bound DNA, 13 µL of PE1.0 PCR primer (1.5 pmol), 13 µL PE2.0 PCR primer (1.5 pmol), 100 µL 2x Phusion High Fidelity master mix, and 34 µL of water for a total of 200 µL. For verification 1% of the PCR was kept and the remainder was purified using the Qiagen PCR Minelute kit per the manufacturer’s protocol eluting twice with 1x TLE. A 2% agarose gel was ran to ensure that the large scale PCR is the correct size range and sufficient for sequencing. The sequencing was done on a Genome analyzer II with one or two lane(s) of 2x120 by the University of Missouri DNA core.

**Patient samples**

Patient samples were obtained from the Ellis Fischel Cancer Center (Columbia MO) following diagnostic evaluation in compliance with the local Institutional Review Board.

**Cell CµLture**

Human cell lines RL and MHH-Call-4 were maintained at 37^o^C with 5% CO_2_. MHH-Call-4 is a human B cell precursor leukemia cell line established from the peripheral blood of a 10-year-old Caucasian boy with acute lymphoblastic leukemia at diagnosis in 1993 (Tomeczkowski et al., Br J Haematol 89: 771-779 (1995). It was grown in DMEM with 20% heat inactivated FBS and split twice a week. RL is a human B cell lymphoma cell line established in 1983 from the ascites of a 52-year-old with B-NHL (Beckwith et al., 1990). It was grown in DMEM with 10% FBS and split twice a week.
